# Spatially resolved profiling of steroid nuclear receptors reveals a role for the disordered N-terminal domains in genome targeting and AP-1 interaction

**DOI:** 10.1101/gr.280896.125

**Published:** 2026-07

**Authors:** Inbal Zigdon, Achinoam Shoham, Vladimir Mindel, Wajd Manadre, David Jan, Beniamin Krupkin, Dekel Yahav Har-Shai, Yaron Antebi, Naama Barkai

**Affiliations:** Department of Molecular Genetics, Weizmann Institute of Science, Rehovot 76100, Israel

## Abstract

Transcription factors (TFs) regulate gene expression by binding to *cis*-regulatory regions that contain their specific binding motifs. In budding yeast, intrinsically disordered regions (IDRs) have emerged as key effectors of genome preferences; however, whether this principle holds in the complex mammalian genomes remains unclear. Here, we profile the binding of three nuclear steroid receptors (SRs) containing long disordered N-terminal domains (NTDs), confirming that the NTDs direct SR binding across genomes. Motif enrichment, coupled with footprint analysis, reveals that SR mutants lacking their NTDs favor binding in proximity to the AP-1 motif, whereas the NTD permits binding at the canonical SR motif also when distant from AP-1 sites. Furthermore, profiling SRs and their mutants in budding yeast confirms that the NTD can direct genome targeting also in the absence of its mammalian-specific interactors. Our results provide new insights into the specificity of SR target selection, opening new avenues for the potential modulation of their activities.

Transcription factors (TFs) contain DNA-binding domains (DBDs) that bind to selected DNA sequence motifs. However, these motifs are too short and abundant to explain the TF locations across vast genomes. Besides DBDs, eukaryotic TFs are characterized by extensive intrinsically disordered regions (IDRs) that span hundreds of amino acids (AAs) (Ward et al. 2004; Liu et al. 2006; Minezaki et al. 2006; Peng et al. 2014; van der Lee et al. 2014; Skupien-Rabian et al. 2016; Wang et al. 2016; Ferrie et al. 2022; Holehouse and Kragelund 2024; Jonas et al. 2025). IDRs remain understudied because they are challenging to purify and because their unique sequence-function relationships elude sequence alignment, requiring new approaches for comparative sequence analysis (Riback et al. 2017; Zarin et al. 2019; Amin et al. 2020; Bugge et al. 2020; Choi et al. 2020; Cohan et al. 2022; Langstein-Skora et al. 2026).

Our previous studies of budding yeast TFs showed that IDRs can direct TFs to their genomic target sites (Brodsky et al. 2020, 2021; Jana et al. 2021; Rosenblum et al. 2021; Kumar et al. 2023; Hurieva et al. 2024), which was perhaps unexpected given that specific binding is commonly attributed to structural complementarity. We provided evidence that IDRs direct genome binding through multiplicity of weak binding determinants (Brodsky et al. 2020; Kumar et al. 2023; Mindel et al. 2024a) and showed that IDR-favored promoters share a common nucleosome architecture (also termed OPN) (Tirosh and Barkai 2008), contrasting the DBDs, which bound their motifs across all promoter types (Kumar et al. 2023; Mindel et al. 2024b). Mechanistically, IDRs could direct genome binding by interacting with cobinding TFs or with nucleosomes (Ferrie et al. 2022; Staller 2022). However, both models were challenged by our studies of the Msn2 TF (Jonas et al. 2023; Lupo et al. 2023; Jana Lang et al. 2024; Mindel et al. 2024a), potentially pointing to a novel IDR–DNA recognition paradigm that differs from the precise, structure-based DNA associations offered by DBDs (Jonas et al. 2025).

Compared with budding yeast, higher eukaryotes are characterized by larger genomes and expanded TF repertoires (about 150 in yeast vs. about 1500 in human TFs) (Weirauch et al. 2014; Lambert et al. 2018). Gene regulation has also evolved to depend on cooperation between proximal promoters and distant enhancers, facilitated by a more complex chromatin architecture and intricate 3D genome folding (Lambert et al. 2018; Kim and Wysocka 2023). Chromatin-mediated restriction of TF binding and combinatorial cobinding of interacting TFs may play more prominent roles in these genomes compared with budding yeast (Kim and Wysocka 2023). It is, therefore, unclear whether principles governing the TF binding preferences in budding yeast, including the use of IDRs, apply to higher eukaryotes, and if so, how they have evolved to accommodate the increased complexity and higher regulatory demands.

Contrasting the large expansion in genome sizes and regulatory complexity, the sequence architecture of the TFs themselves remains conserved across eukaryotes (Lambert et al. 2018). Conserved TF features include common DBD families of shared DNA motifs and the abundance of long IDRs outside the DBDs (Jonas et al. 2025). Indications that IDRs may direct TF bindings across mammalian genomes were provided by live tracking of single TFs, which revealed IDR-dependent chromatin associations, and by genomic analysis that compared binding locations of TFs and their IDR mutants (Burdach et al. 2014; Boller et al. 2016; Garcia et al. 2021; Chen et al. 2022; Ferrie et al. 2022; Lerner et al. 2023; Mazzocca et al. 2023; Naderi et al. 2024; Zolotarev et al. 2024; Lambourne et al. 2025). Also of interest is the prevalence of de novo DBD–IDR fusions within oncogenic proteins, including the gaining of new target binding sites by EWS–FLI1, a major oncogene in Ewing's sarcoma (Gangwal et al. 2008; Grünewald et al. 2015; Boulay et al. 2017; Chong et al. 2018, 2022).

Motivated by these findings, we set out to test the role of IDRs in directing genome binding of mammalian TFs. As a case in point, we selected three nuclear steroid receptors (SRs) that share a conserved DBD and disordered N-terminal domains (NTDs): the androgen receptor (AR), nuclear receptor subfamily 3, group C, member 1 (NR3C1, also known as the glucocorticoid receptor [GR]), and progesterone receptor (PGR, also known as PR). In this selection, we were motivated by the lengths of the disordered NTDs (417–560 AAs) and the fact that the three share the same consensus DBD-binding motifs while regulating distinct functions. Furthermore, for one of the three (GR), deletion of the disordered NTD was previously shown to reduce chromatin confinement and abolish binding at a large fraction of genomic sites (Garcia et al. 2021). To define the TF binding sites at footprint-level spatial resolution, we optimized ChEC-seq (Schmid et al. 2004; Zentner et al. 2015) for mammalian genomes, enabling the detection of TF footprints within binding peaks and the inference of motif usage and cobinding interactions.

## Results

### Using ChEC-seq for mapping TF binding locations across mammalian genomes

**Figure 1.**
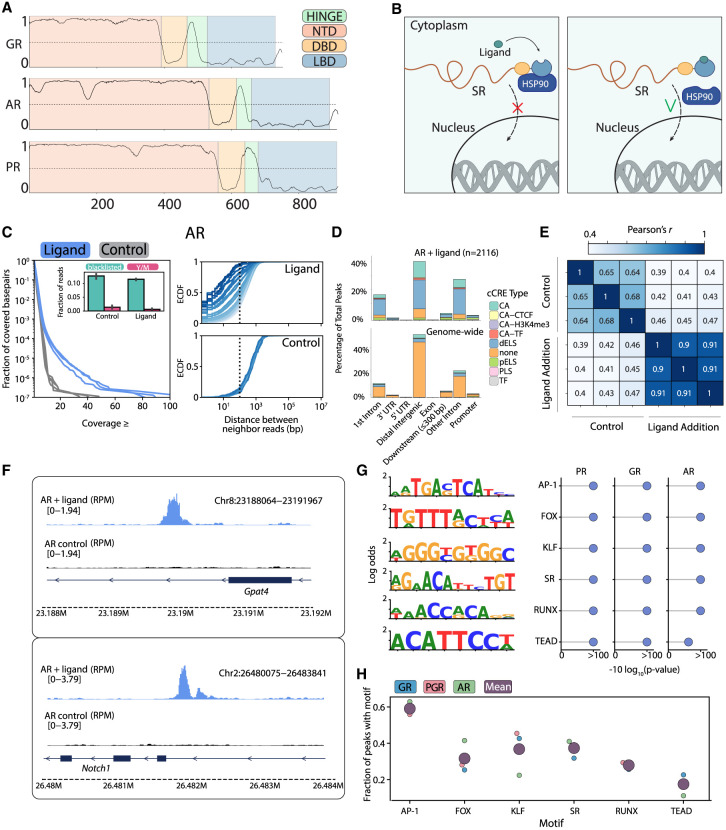
Using ChEC-seq for mapping transcription factor (TF) binding locations across mammalian genomes. (*A*) The domain architecture of the three selected SRs. The predicted protein-sequence disorder tendencies of GR, AR, and PR are shown using Metapredict V2 (Emenecker et al. 2021). The four domains characterizing the NR TFs are color-coded, as indicated. (*B*) Scheme of how hormone binding releases SR from cytoplasmic retention. The binding of hormones to the ligand-binding domain (LBD) releases the SRs from cytoplasmic retention by the HSP90 chaperone, triggering their nuclear translocation, when they bind and regulate their target genes. (*C*) ChEC-seq profiles of activated SRs are distinguished by the distribution of cleaved sites. Shown are the distributions of per-nucleotide reads coverage for hormone-stimulated and nonstimulated AR (*left*; blue and gray lines, with three repeats each; Methods) and the distribution of distances between cleavage sites (*right*; with cleavage events grouped by read coverage indicated by color intensity; Methods). Note that cleavage sites are sparsely distributed in the control sample but tend to accumulate at the same or adjacent reads in the stimulated ones. The fraction of reads that map to the blacklisted regions and the Y or mitochondrial chromosomes is shown in the *insets*. For similar data for GR, PR, and SMAD1, see Supplemental Figure S1C. (*D*) SR binding peaks preferentially overlap regulatory sites. Shown on the *left* is the fraction of AR binding peaks localized across genomic features and *cis*-regulatory elements (CRE), compared with their genomic distributions. Here, SCREEN (The ENCODE Project Consortium et al. 2020) annotations were used for distal or enhancers, proximal enhancers, or regions lacking annotations (“dELS,” “pELS,” and “none”). (*E*) Binding profiles of SRs are reproducible. Correlation in peak binding across repeats of hormone-stimulated and nonstimulated controls (Methods). (*F*) Genomic tracks of AR binding. Shown are *Gpat4* and *Notch1*, selected as strong targets of AR. Note the strong binding signal specifically of the hormone-stimulated AR. (*G*) Motif enrichment within SR peaks. Shown are the top enriched motifs within the three SR-bound peaks, as found by de novo motif finders, together with the respective *P*-values (Methods). (*H*) The fraction of peaks containing each motif. Shown for the individual SRs (small dots) or the average of the three (purple dot).

The nuclear SRs belong to the nuclear receptors (NRs) family of TFs, whose common domain architecture includes a centrally located zinc-finger DBD, a C-terminal ligand-binding domain (LBD), and a disordered NTD (Fig. 1A; Mangelsdorf et al. 1995; Kininis and Kraus 2008; Gustafsson 2016; Weikum et al. 2018). The SR subfamily contains six members that are activated by cholesterol derivatives. The binding of a ligand to the LBD releases the SR from cytoplasmic retention and triggers nuclear localization and DNA binding (Fig. 1B; Grad and Picard 2007). The three SRs we selected (AR, GR, and PR) share conserved DBDs that bind as homodimers to a common motif composed of two inverted 6-bp sequences (AGAACA) separated by 3 bp of random composition (Laudet et al. 1992).

To map genomic locations, we optimized ChEC-seq (Zentner et al. 2017) for mammalian cells. In ChEC-seq, the TF of interest is fused to micrococcal nuclease (MNase), which enables a cleavage of TF-proximal DNA upon a brief calcium pulse. The cleaved DNA fragments are collected, sequenced, and mapped to the genome, yielding TF binding maps at a spatial resolution that approaches a single base pair (Supplemental Fig. S1A). We favor this method as it is highly sensitive, independent of antibodies or cross-linking, and requires relatively low sequencing depth (10 m reads).

As a convenient cellular model that is not specifically associated with the selected SRs, we chose the mouse mammary gland cell line (NMuMG). The SRs are tightly conserved between mice and humans, so for technical reasons, we selected the murine AR and human PR/GR (Supplemental Fig. S1B). We fused each SR to an MNase, cloned this fusion downstream from a *Pgk1* promoter, and introduced it into the genome using the piggyBac transposon system (Methods). We then grew cells to densities of about 1.4 million and profiled samples before and after hormone stimulation. Sequenced reads were aligned to the mm10 genome, and blacklisted regions were removed following standard practice (Methods). Note that in the nonstimulated cells, the SRs are expected to remain cytoplasmic and show little, if any, DNA binding signal. SMAD1, a ligand-dependent TF differing in motif preferences, was included as an additional control.

ChEC-seq profiles of hormone-stimulated SRs were distinguished from the nonstimulated controls in showing a pronounced accumulation of cutting sites at the same or closely spaced nucleotides (Fig. 1C; Supplemental Fig. S1C,D). Furthermore, binding peaks (Methods) were enriched in regulatory and gene-proximal regions, reproduced in independent repeats (c > 0.9) (Fig. 1D,E; Supplemental Fig. S1E), and associated with known regulatory functions (Fig. 1F; Supplemental Fig. S1F). ChEC-seq was sensitive enough to detect the residual activation of SMAD1 by the residual levels of its BMP4 ligand present in our freshly added growth media (Supplemental Fig. S1C, center).

De novo motif analysis revealed that SR binding peaks were enriched with a common set of motifs (Fig. 1G; Supplemental Fig. S2A). Top-enriched motifs include the canonical SR binding site, which was present in ∼40% of bound peaks, together with motifs of AP-1, FOX, KLF, RUNX, and TEAD (∼60%, 30%, 40%, 25%, and 20% of peaks, respectively) (Fig. 1H). Each of these noncanonical motifs was identified in previous analysis of at least one SR in various cell lines (Jonat et al. 1990; Herrlich 2001; Reddy et al. 2009; Biddie et al. 2011; Clarke and Graham 2012; Mazur et al. 2015; Wilson et al. 2016; Dinh et al. 2019; Ogara et al. 2019; Paakinaho et al. 2019; Garcia et al. 2021; Grbesa et al. 2021; Johnson et al. 2021; Wissink et al. 2021). Binding peaks were typically ∼300–600 nucleotides (nt) and contained one to three distinct motifs, with coassociation of AP-1 and SR being the most frequent (Supplemental Fig. S2B–D). Together, these results support the use of ChEC-seq for profiling TF binding across mammalian genomes.

### Differences in SR binding sites extend beyond hormone–LBD effects and contrast conserved binding preferences of their DBDs

**Figure 2.**
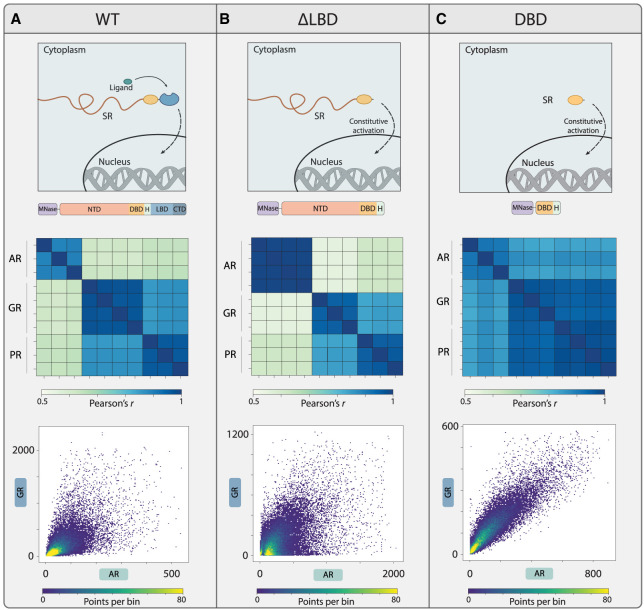
Differences in SR binding profiles are not explained by LBD or DBD preferences. (*A*) AR binding peaks differ from those of GR and PR. Binding profiles of the hormone-stimulated SRs were compared (*top*; scheme, illustrated *beneath* are the full-length MNase-fused SRs). Shown is the correlation of binding signals across the set of peaks bound by at least one of the compared factors (about 30,000 peaks), with all repeats included (beneath; Methods). Binding at individual peaks is also compared as scatter plots (*bottom*; sum of signal on peak; 99.8% of data is shown to remove the outliers, and the color indicates density). (*B*) Binding locations of LBD-deleted AR are distinct from those of GR and PR. Hormone-independent activation of SRs was achieved by deletion of their ligand-binding domains (LBDs). Binding profiles were measured in the absence of hormones (*top*; scheme, illustrated *beneath* are the LBD-deleted mutants). The figure compares the binding profiles of the three LBD-deleted mutants, as in *A*. (*C*) The three DBDs bind the same genomic sites. DBD mutants were generated by deleting both the LBD and the NTD. Binding profiles were measured in the absence of hormones (scheme, illustrated *beneath* are the DBD mutants). The figure compares the binding profiles of the three DBD mutants, as in *A*.

The binding peaks of all three SRs were therefore enriched with the same set of motifs. However, the binding peaks themselves differed substantially between AR and GR or PR (Fig. 2A; Supplemental Fig. S3A). As a first explanation for these differences, we considered hormone exposure, because the three SRs were activated by distinct hormones. To mitigate this, we deleted the LBD, which alleviated cytoplasmic retention, leading to activation and DNA binding even in the absence of hormones (Fig. 2B, top; Dehm and Tindall 2011; Chan and Dehm 2014). All three LBD-deleted SRs (SR^ΔLBD^) displayed robust DNA binding, as confirmed by the criteria described above (Supplemental Fig. S3B,C). Notably, although bindings were profiled in the absence of hormones, binding peaks of AR^ΔLBD^ were still substantially different from those of GR^ΔLBD^ or PR^ΔLBD^ (Fig. 2B; Supplemental Fig. S3D).

To test whether these differences in binding preferences can be explained by the respective DBDs, we generated DBD-only mutants (SR^DBD^) lacking both the LBD and the NTD (Fig. 2C, top; note that the DBD-dimerization domain was retained). The three DBDs localized to a highly similar subset of peaks (correlation = 0.85–0.9, comparable to replicate concordance of 0.9–1) (Fig. 2C). We conclude that the three SRs have distinct binding preferences, which are not driven by their DBDs, as all three DBDs appear functionally equivalent. This pointed to the disordered NTD as a source of binding preference between the SRs.

### The disordered NTD directs binding preferences of AR and PR using a multiplicity of weak determinants

**Figure 3.**
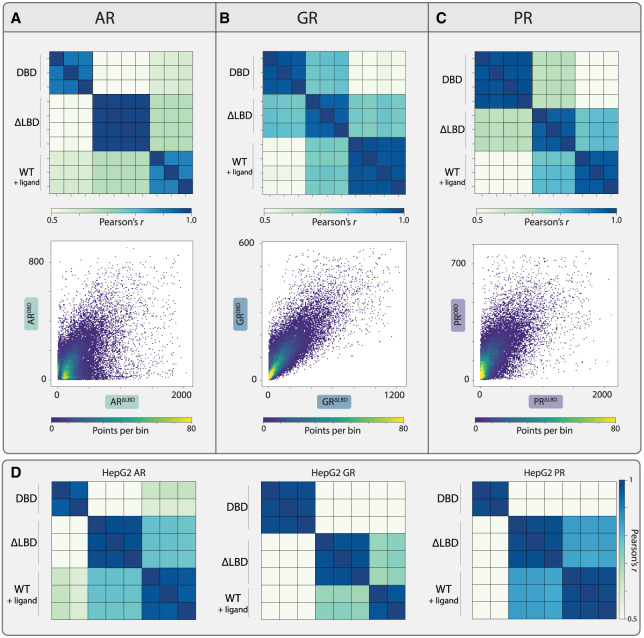
Both the NTD and the LBD contribute to the selection of SR binding targets. (*A*–*C*) NTD deletion shifts the binding preferences of SRs. Shown are the correlations in peak binding between each SR and its LBD-deleted and DBD mutants, with all repeats included (*top*). Binding at individual peaks is also compared as scatter plots (*bottom*; sum of signal on the peak, and the color indicates density). (*D*) The NTD directs SR binding in the HepG2 cell line. We profiled binding locations of the three SRs and the indicated mutants in the HepG2 cell line. The correlations in peak binding between the full-length SRs and the indicated mutants are shown.

We next tested more directly the influence of the disordered NTD and the LBD of SRs on binding by comparing the binding peaks of each SR with those of its SR^ΔLBD^ and SR^DBD^ mutants. As expected, LBD deletion shifted binding locations, likely resulting from LBD-mediated cofactor interactions, hormone-specific effects, or LBD–NTD interaction. Binding preferences were also shifted when deleting the NTD, both in the background of LBD-deleted mutants (Fig. 3A–C; Supplemental Fig. S3E) and the full-length SRs (containing the LBD) (Supplemental Fig. S4). Therefore, to study the influences of the NTD on genome targeting, we decided to focus on the LBD-deleted SRs to reduce the confounding effects of LBD–hormone interactions or LBD–NTD interactions.

NTD deletions substantially altered the binding of AR and PR but had a weaker effect in the case of GR (Fig. 3A–C; Supplemental Figs. S3E, S4). Earlier results using a different cell line showed a strong dependency of GR binding on its NTD (Garcia et al. 2021). We therefore repeated our experiment using a second cell line, HepG2 (Supplemental Fig. S5A–C). In this background, NTD deletion altered the binding of all three SRs (Fig. 3D; Supplemental Fig. S6A). The possible explanation for the difference in the NTD-deletion effect for the GR could arise from the different cofactors expressed in the two cell lines. Notably, although NTD deletion caused loss of binding peaks (coupled with a gain of others) (Supplemental Fig. S3E), protein abundance was consistently increased by these deletions, as captured in western blot analysis (Supplemental Fig. S6B). We conclude that the NTD of SRs can influence their genomic target selection, independent of LBD interaction or hormonal stimulation.

**Figure 4.**
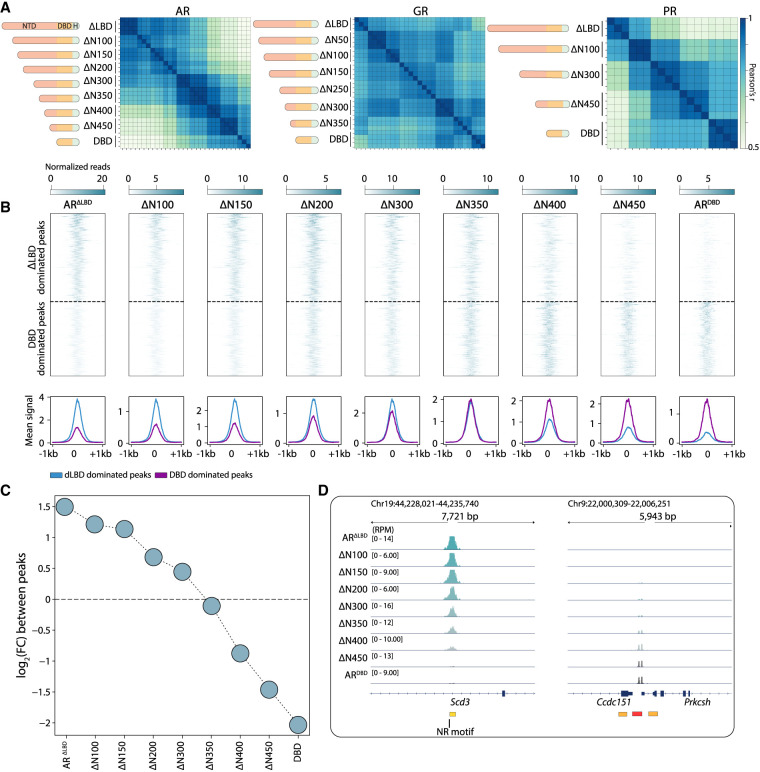
Multiple weak determinants spread across the SR NTD sequence guide their binding specificity in mammalian genomes. (*A*) A NTD truncation series reveals that the binding profiles of AR and PR are gradually shifted with increasing length. The NTDs were gradually shortened by removing defined amino acid (AA) blocks, as shown in the schemes. Shown are the correlations in peak binding between the indicated truncation mutants (note that all repeats are included). (*B*–*D*) Truncations of the NTD of AR result in a gradual loss of binding peaks. (*B*) The gradual loss of LBD-deleted mutant-dominated peaks and gain of DBD mutant-dominated peaks upon NTD truncations (*top*; Methods), as well as the mean signal of the dominated peaks of the two mutants (*bottom*). (*C*,*D*) Log_2_ fold-change of signal on the ΔLBD-dominated peaks versus DBD-dominated peaks, as defined above, is shown in *C*, and two genomic tracks are shown as a representative example of the loss of LBD-deleted (ΔLBD) mutant peaks and gain of DBD mutant peaks (*D*; Methods).

In budding yeast, IDR-directed TF binding often depends on a multiplicity of weak determinants spread across the sequence (Brodsky et al. 2020; Kumar et al. 2023; Hurieva et al. 2024). To assess whether this is also the case for the SR NTDs, we generated a series of truncation mutants by sequentially removing AA blocks from the N terminus of each SR (Fig. 4A). Also here, we favored using an SR mutant lacking its LBD to alleviate hormonal effects or possible NTD–LBD interactions. For AR and PR, the truncations gradually shifted binding preferences with increasing truncation length, as evidenced by peak correlation patterns (Fig. 4A), a transition toward DBD-like peak dominance (Fig. 4B,C; Supplemental Fig. S7A–C), and the binding signals across the individual peaks (Fig. 4D). We conclude that the NTD plays a central role in directing SR genomic binding, acting through multiple determinants distributed throughout its sequence.

### NTD truncations do not change motif preferences but alter footprint signatures

**Figure 5.**
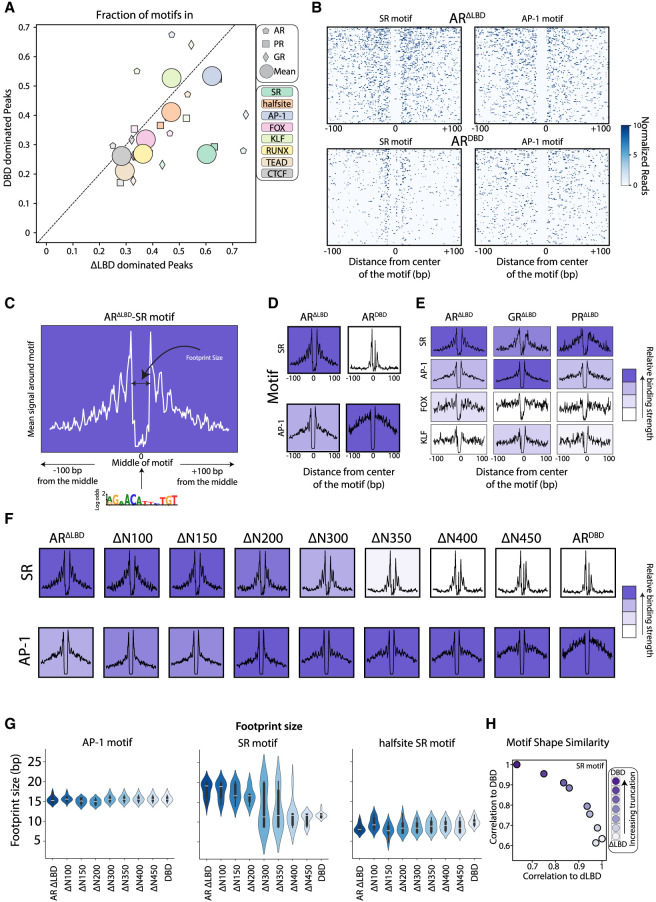
Footprint analysis defines SR binding at canonical and noncanonical motifs. (*A*) Enrichment of noncanonical motif remains largely invariant to NTD deletion. Shown are the fractions of motifs in SR^ΔLBD^-dominated (*x*-axis) and SR^DBD^-dominated (*y*-axis) peaks (as defined in Fig. 4) for the eight analyzed motifs and three SRs, as indicated. The circles indicate the mean over the three SRs. (*B*–*D*) Visualizing the SR and AP-1 footprint. (*B*) The locations of cleavage sites (read ends) of the indicated factors at sequences surrounding the indicated motifs (Methods). For this analysis, the top 10% bound motifs within binding peaks were selected, ordered by the binding signal, and are shown as rows in this matrix, together with the ±100 bp surrounding regions (Methods). (*C*,*D*) The annotated example of the average profile for the AR^ΔLBD^ on the SR motif (*C*); respectively, mean profiles for the indicated AR mutants and motifs (*D*). The background is colored by the mean signal at the motif and normalized intrafactor (column-wise). Note the virtually absence of reads at the motif itself, and the accumulation of reads at the immediate flanking regions. (*E–H*) Truncation of NTD and the motif signatures of SRs. (*E*) The average profiles of LBD-deleted mutants over the indicated motifs. (*F*) The average profiles of the AR truncations on the SR and AP-1, background color as in *D*. (*G*) The estimated sizes of the cleavage-protected region (footprint size; Methods) for the AR variants for AP-1, SR, and half-site motifs. (*H*) Correlation of SR motif signal to DBD reference motif (*y*-axis) versus ΔLBD reference motif (*x*-axis) for each truncation variant. Color gradient indicates progressive truncation from ΔLBD (white) to maximally truncated DBD-only (dark purple) as indicated in the legend.

The disordered NTDs could direct SR binding by interacting with cobinding TFs. Potential candidates include TFs that bind the noncanonical motifs enriched within the SR-bound peaks. However, we noted that all these noncanonical motifs were enriched not only in peaks of the LBD-deleted mutants but also in peaks bound by the mutants lacking the NTD, SR^DBD^ (Fig. 5A; Supplemental Fig. S8A). Furthermore, the frequency of these motifs within the binding peaks was largely invariant to NTD truncations; The AP-1 motif, for example, was present consistently within ∼60% of bound peaks across the NTD truncation series (Supplemental Fig. S8B). Of note, SR^DBD^ binding peaks were comparable in size to the full SRs peaks, spreading from 300 to 700 nt (Supplemental Figs. S8C, S2B).

A major advantage of ChEC-seq is the spatially resolved data that enable detecting TF footprints: The TF-bound DNA itself is protected from MNase cleavage, and high cleavage is detected at its immediate flanking regions. SR footprints were evident when plotting the read ends around bound SR motifs in peaks (Fig. 5B,C; Supplemental Fig. S8D). Clear footprints were also seen at the noncanonical AP-1 motif within bound peaks (Fig. 5B; Supplemental Fig. S8D). It is notable that the footprint on the AP-1 motif was seen in both the LBD-deleted and truncated SR strains (Fig. 5D) and displayed both footprint features: First, the motif itself was protected from cleavage, which indicates a binding event, either of only AP-1 or also of SR. The second feature was the strong binding peaks at the borders of the AP-1 motif (Fig. 5D). This provided a strong indication that AP-1 sites not only are protected owing to AP-1 DNA occupancy but also serve for the recruitment of SR themselves. Of note, recruitment of SR to the AP-1 motif is consistent with the known “tethering” of GR to the AP-1 motif, either through interaction with the AP-1 complex or through direct binding to the AP-1 motif (Yang-Yen et al. 1990; Reichardt et al. 1998; Ogawa et al. 2005; Cain and Cidlowski 2017). At the other enriched motifs, the footprint appeared weaker (e.g., FOX) or less consistent across the three factors (e.g., KLF) (Fig. 5E). In our control SMAD1 data, footprint signals were again seen not only at the SMAD motif but also at the AP-1 motif, consistent with the known interaction of SMAD1 with AP-1 (Liberati et al. 1999). In this case, SMAD data showed no footprint at the SR binding motif, confirming the specificity of the footprints (Supplemental Fig. S8E).

Repeating our footprint analysis for all truncation mutants showed a transition toward an AP-1 dominant footprint with increasing truncation (Fig. 5F). The size and pattern of the TF footprint depend on the bound complex, which may differ between motifs or SR mutants. To capture that, we examined more closely the footprints at the AP-1 and SR motifs across the three different SRs and the respective NTD truncation series. The signatures were consistent across the three SRs (Fig. 5F–H; Supplemental Figs. S8F, S9A,B), but we noted that both the size of the SR binding signatures and their binding strengths varied with increasing truncations. First, although the size of AP-1 footprints was consistently ∼15 bp across all truncations, the size of the footprint at the canonical SR motif decreased from ∼20 bp to about half its size in the NTD-truncated mutants (Fig. 5G; Supplemental Fig. S8F). Second, the binding strength at the SR motif was decreased with longer truncation, whereas the binding strength at the AP-1 motif was increased (Fig. 5F; Supplemental Fig. S9A). We conclude that ChEC-seq data provide spatially resolved data enabling TF footprint visualization. According to this analysis, the NTD does not appear to direct target selection by enabling interactions with TFs that localize to the noncanonical motifs enriched in the SR-bound peaks.

### SR mutants lacking the NTD show tighter localization to the AP-1 motif

**Figure 6.**
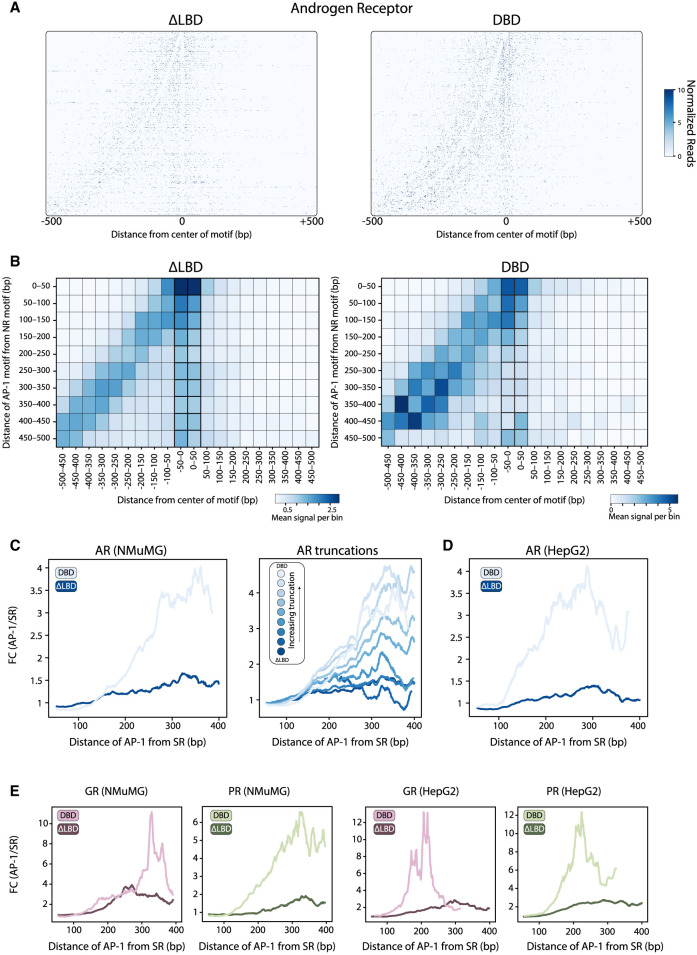
Binding of the SR^DBD^ mutants is stronger at the proximal AP-1 motif than at the SR motif itself. (*A*) Visualizing the signal-footprints of adjacent motifs. Genomic regions from the peaks containing both SR (canonical) and AP-1 motifs (noncanonical) were aligned to the SR motif center and sorted by intermotif distance, with AP-1 positioned upstream for AR^ΔLBD^ (*left*) and AR^DBD^ (*right*). The heatmap shows MNase-cleaved fragment ends, revealing protection patterns at both motif sites. (*B*–*E*) Proximity to AP-1 motif increases binding of AR^DBD^ to its canonical motif. (*B*) The bindings of AR^ΔLBD^ (*left*) and AR^DBD^ (*right*) binned by distance from SR center (*x*-axis, 50 bp) and AP1-to-SR spacing (*y*-axis, 50 bp). The color scale shows mean normalized reads per bin. (*C*, *left*) The distance-dependent effect is summarized, showing the fold-change of the signal at the AP-1 versus SR motifs as a function of the distances between motifs. The same analysis is also shown for the set of AR truncations (*C*, *right*) and for two mutants of the AR in the HepG2 cell line (*D*). (*E*) The distance-dependent effect for the GR and PR mutants in NMuMG and HepG2 cell lines.

To examine for potential NTD-mediated interactions in more detail, we extended our footprint analysis to examine the binding of SR within peaks containing its canonical and noncanonical motifs. We reasoned that if the NTD promotes TF interactions, it would favor sites containing nearby other-TF motifs, compared with the DBD-only mutants. For this, we first asked whether binding at noncanonical motifs within peaks is biased toward the canonical SR motif. We grouped all peaks containing the SR motif together with a given second motif (e.g., AP-1), ordered the sequences by the intermotif distances, and aligned them by the SR motif, placing the second motif to the left. Notably, contrasting with our expectation, clear footprints were observed at both the canonical and noncanonical motifs (Fig. 6A; Supplemental Fig. S10A). The observed pattern suggested that SR binds to both its canonical and the AP-1 motifs within the same binding peaks (Fig. 6A; Supplemental Fig. S10A). In fact, comparing more specifically whether SR^ΔLBD^ binding at its own motif decreases with the distance from the AP-1 motif revealed that binding at both motifs appeared largely invariant to the distance between them (Fig. 6B, AP-1; Supplemental Fig. S10B).

Next, we tested whether deletion of the NTD alters the binding of SR^DBD^ at the AP-1 motif. We expected weakening of the binding signal at distant AP-1 motif occurrences; however, the deletion of the NTD did not reduce binding at AP-1 sites but rather increased preference to this motif. In fact, binding at the canonical SR motif was seen primarily at proximity to the AP-1 motif but decreased with higher intermotif distances (Fig. 6B–E; Supplemental Fig. S10B,C). This effect was seen in all three SRs and in the two cell types we tested. The only exception was GR in NMuMG cells that displayed favored binding at proximity to the AP-1 site even in the presence of the NTD (Fig. 6E; Supplemental Fig. S10C, left). Furthermore, the apparent favored binding at the proximal AP-1 motif strengthened gradually with increasing NTD truncations (Fig. 6C; Supplemental Fig. S10C). Therefore, the NTD contributes to SR binding preference in at least two, possibly related manners: first, by selecting the location of binding peaks and, second, by alleviating the strong DBD bias toward the AP-1 motif, thereby enabling binding at the canonical SR motif within these peaks.

### The binding of SRs across the budding yeast genome depends on their disordered NTDs

**Figure 7.**
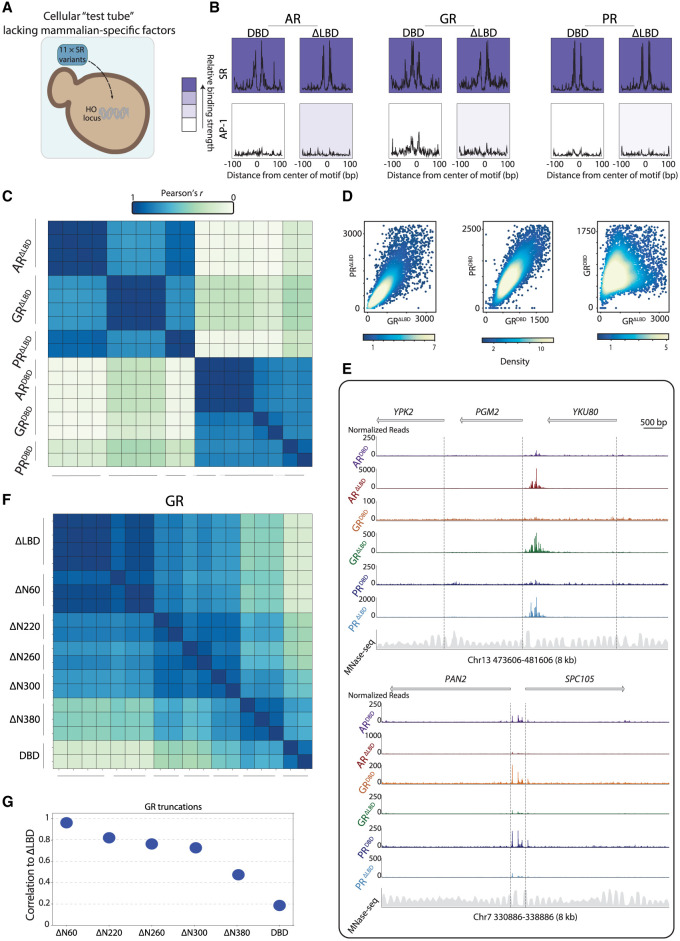
NTD directs SR binding across the budding yeast genome. (*A*) Using yeast as a “cellular test tube” to study NTD effects on the genome binding preferences of SRs. The three selected LBD-deleted SRs (ΔLBD) and the respective DBD-only mutants (DBD) were introduced into the yeast genome, and their binding profiles across the genome were measured using ChEC-seq. (*B*) Motif binding by LBD-deleted SRs and their DBD mutants. The average profiles of the indicated factors around mammalian-enriched motifs are shown at the indicated motifs; background color is as in Figure 5D. (*C*–*E*) The NTD directs SR binding preferences across the budding yeast genome. Shown are the correlations in promoter preferences of the indicated SR mutants (Methods), including multiple independent replicates (*C*), as well as respective scatter plots (showing here data in the Q1/Q3 ± 1.5 × IQR range), comparing promoter binding of the indicated strains in scatters (*D*) and representative binding tracks (*E*). (*F*,*G*) GR binding preferences shift gradually with increasing NTD truncation. The LBD-deleted GR truncation series was introduced into budding yeast, and their binding profiles were measured. The correlation matrix across all truncations (*F*) and the correlations of promoter preferences of the indicated truncations to the untruncated GR ΔLBD mutant (*G*) are shown.

The finding that NTDs did not increase the apparent dependency of SR binding on noncanonical motifs challenges the cooperative binding model as the basis for their role in genome recognition. To examine this further, we asked whether the NTDs would influence binding if tested in a system that lacks mammalian-specific cofactors (Fig. 7A). We profiled the binding of SR^ΔLBD^ and SR^DBD^ in yeast cells (Methods). All six factors localized preferentially to the SR motif but showed no enrichment or footprint at the AP-1 motif (Fig. 7B). Of note, in this system, we observed little, if any, binding at the SR half-site, and footprint size had a high variance regardless of NTD deletion (Supplemental Fig. S11A,B).

In yeast, all three SR^DBD^ localized at practically the same sites; however, these favored sites were distinct from the locations bound by the three SR^ΔLBD^ (Fig. 7C–E). Thus, also in budding yeast, the disordered NTD directed SR binding across the genome, and this was observed for all three SRs. In this, NTDs steered binding toward OPN-type promoters, which are favorably bound by budding yeast TFs (Supplemental Fig. S11C; Tirosh and Barkai 2008; Kumar et al. 2023). To test whether the NTDs are (accidentally) recruited by native yeast TFs, we considered our compendium of 159 binding profiles of native yeast TFs, which were all measured under the same conditions (Brodsky et al. 2020; Gera et al. 2022; Kumar et al. 2023; Lupo et al. 2023). Notably, the maximal correlations between promoter preferences of the native TFs and the SR^ΔLBD^ were rather low, reaching only *c* = 0.6 (Supplemental Fig. S11D). We conclude that the NTD directs SR binding to distinct sites across the budding yeast genome.

Finally, we asked whether, in budding yeast, the NTDs also act through a multiplicity of determinants. Indeed, the GR NTD truncation series displayed a gradual shift in binding preferences (Fig. 7F,G). We conclude that, as in the mammalian genome, the NTD governs binding preferences across the budding yeast genome, using a multiplicity of weak binding determinants.

## Discussion

TFs of the NR family contain a characteristic disordered NTD. In this study, we report that these NTDs can assist in directing genome recognition in both the presence and absence of the LBD. We focused on three closely related SRs, including the ARs, GRs, and PRs, whose 417–560 AA (disordered) NTDs are among the longest in the NR family. Central to our approach is the use of mammalian-optimized ChEC-seq, which enables high-resolution profiling of genome-wide binding. This allowed us to analyze not only the locations of binding peaks but also motif-specific footprints and interactions within individual binding regions. In characterizing the NTD effects, we focused primarily on LBD-deleted SR mutants, which are constitutively nuclear-localized and, therefore, bind DNA also in the absence of the activating hormones.

We find that the disordered NTD acts both in selecting the SR binding peaks and in biasing binding between motifs within these peaks. We observe the changes in peak preferences for the three SRs we tested, although the extent of NTD contribution differed between the factors and cell lines. In the murine NMuMG cells, sensitivity to NTD deletion was greater for AR and PR than for GR, whereas in the HepG2 cell line, the binding of all three SRs was perturbed by this deletion. Furthermore, in AR and PR, shortening the NTD gradually through a series of truncations led to a gradual shift in peak selection, attributing the NTD activity to a multiplicity of weak determinants, as seen in budding yeast TFs.

In addition to directing the selection of binding peaks, the NTDs also changed the pattern of motif binding within peaks but in a way that appeared counterintuitive to us at first. We initially expected NTD deletion to reduce SR binding at noncanonical motifs in accordance with the prevailing model of cobinding interactions. However, in contrast to this expectation, NTD deletion increased, rather than decreased, binding at the AP-1 motif and further decreased binding at the canonical (isolated) SR motif itself. The AP-1 motif was highly prominent, found in 60% of peaks. Our footprint analysis indicated that it was bound by the untruncated SRs at levels comparable to the canonical motifs through all distances between the two motifs. The AP-1 motif also remained strongly bound upon NTD deletion, and SR^DBD^ mutants were localized to their canonical motif only if it was proximal to an AP-1 one.

The association of GR with the AP-1 motif was described previously and is of great interest, given its centrality to the GR repression of inflammatory genes (Karin 1998; Reichardt et al. 1998). In the literature, two models were proposed to explain this association. First. GR could be tethered by the AP-1-bound TF(s) through direct protein–protein interactions involving the GR and its DBD (Yang-Yen et al. 1990; Reichardt et al. 1998; Ogawa et al. 2005; Cain and Cidlowski 2017). Second, GR could recognize the AP-1 motif even in the absence of the AP-1 TFs, as it can bind as a monomer to a proxy of its half-site presented in the AP-1 motif (Weikum et al. 2017; Escoter-Torres et al. 2020). Although both models could explain the binding of the SR^DBD^ at the AP-1 motifs, our results are more in line with the former model. First, we noted that the SR footprint at the AP-1 motif was consistently 15 bp, almost twice the 8 bp footprint seen at the SR half-site, suggesting that an additional factor besides the SR monomer is bound at this site. Furthermore, the SR^DBD^ showed no preference for the AP-1 motif when tested in the budding yeast genome lacking the respective AP-1 TFs. Taken together, our results suggest that direct interaction with the AP-1 TFs dominates the binding profile of the SR^DBD^, but this dominance is reversed in the presence of the NTD to enable AP-1 binding at selected canonical motifs. In this context, it is notable that the fraction of reads that are assigned to binding peaks was largely reduced for SR^DBD^ lacking the NTD, suggesting weaker and perhaps more diffuse binding across the genome outside regulatory regions.

Given that our footprint analysis gave no indication that the NTD directs SR binding through interaction with cobinding TFs, we decided to challenge this model further using budding yeast. In this, we asked whether the NTD can also direct binding across the budding yeast genome, lacking mammalian-specific cofactors. All three NTDs directed SR binding when tested in budding yeast, and this activity appeared to rely on the set of weak determinants distributed throughout their sequence. We conclude that the NTD can direct genome binding even in the absence of mammalian-specific cofactors, although it remains unclear whether the same mechanism is at play in both systems.

In conclusion, our findings that NTDs guide SR binding across both mammalian and yeast genomes have broad implications for understanding TF function and genome-targeting mechanisms. Previous studies from our laboratory have shown that disordered NTDs play a key role in genomic targeting across a substantial group of yeast TFs. Together with the results presented here, this suggests that IDR-directed targeting may represent a more general mechanism of TF targeting not only in yeast but in mammalian systems as well, warranting further investigation. Given the roles of SRs in hormone-driven cancers and other diseases, elucidating the contribution of the NTD to genomic targeting could inform new strategies for rationally modulating SR activity by directing binding to distinct genomic targets.

A limitation of our study follows. Most of our results defining the role of the disordered NTD in directing genomic binding were performed using a mutant deleted of the LBD. We favored this approach as it allowed us to focus on NTD effects that are independent of interactions with the LBD or hormonal effects. However, it should be noted that, by this design, we could not detect LBD-directed interactions (e.g., with cofactors, the NTD of the protein) that are of clear physiological significance to the biology of SRs.

## Methods

### Cloning for the cell culture experiments

Flag-tagged MNase ORF was integrated into Pb510b plasmid (piggyBac transposon system) (Wu et al. 2006) containing a PGK promoter, gifted to us by Dr. Yaron Antebi using restriction ligation with HindIII and XhoI restriction enzymes (NEB R0104S, R0146S) (for the sequence of the plasmid, see Supplemental Table 4). Variant factors ORF were integrated into the above vector (for the sequences of the integrated ORFs, see Supplemental Tables 1–4), using restriction–ligation with XhoI and XbaI restriction enzymes (NEB R0146S, R0145S).

### Cell culture maintenance

All mammalian cell lines (NMuMG and HepG2) were grown in Dulbecco's Modified Eagle Medium (DMEM; without L-glutamine and phenol red, Biological Industries 01-053-1A) supplemented with 1% penicillin–streptomycin (Diagnovum D910), 10% fetal bovine serum (Gibco A5256701), 1 mM sodium pyruvate (Biological Industries 03-042-1B), 1% nonessential AAs (MEM-eagle, Biological Industries 01-340-1B), and 2 mM L-glutamine (Biological Industries 03-020-1B) maintained in a humidifier at 37°C and 5% CO_2_.

### Transfection and generation of stable cell lines

For transfection, cells were plated at a 40% density overnight. Cells were transfected with Dharmafect reagent (DharmaFECT kb, Dharmacon T-2006-01) using the piggyBac transposon system. Following transfection, the selection on the NMuMG cell line was applied using 20 µg/mL hygromycin (Gold Biotechnology H-270-EZ25) and 15 µg/mL hygromycin for HepG2. All cell lines were expanded together with selection. Media was replenished every 48 h with expansion when required. A stable cell line was formed when all cells in nontransfected control died. Cells were counted and plated (between 500,000 and 700,000 cells) 16 h prior to the experiment. On the day of the experiment, full-length variants were treated for 1 h with 100 nM of the appropriate ligand (AR with 5α-androstan, GR with dexamethasone, and PR with progesterone; Sigma-Aldrich A8380, D4902, P8783). Media was replenished for other nontreated control.

### Budding yeast growth, maintenance, and genetic manipulation

All genetic manipulations were performed on the *Saccharomyces cerevisiae* BY4741 strain (genotype: MATa his3Δ1 leu2Δ0 met15Δ0 ura3Δ0 genotype) using CRISPR (Ryan et al. 2016). Transformations were performed using the LiAc/SS DNA/PEG method. After confirming successful transformation with PCR and Sanger sequencing, the pbRA89 (Addgene plasmid 100950) carrying the CRISPR-Cas9 system from positive colonies was lost growing the cells in liquid YPD (yeast extract peptone dextrose) without selection-plating individual colonies and then selecting colonies without bRA89-encoded hygromycin resistance. Ligation of the gene-specific guide-RNA into the bRA89 plasmid was performed as previously described (Ryan et al. 2016).

### Yeast cell growth before experiments

Yeast strains were freshly thawed from frozen stock, plated on YPD plates, and grown. Single colonies were picked and grown in liquid SD medium (synthetic complete with dextrose) overnight at 30°C, reaching stationary phase OD600 ≈ 10, and then diluted again into fresh SD medium for the experiment.

### ChEC-seq experiments

#### Yeast

The experiments were performed as described previously (Zentner et al. 2015), with some modifications. The stationary cultures described above were diluted into 5 mL fresh SD media and grown overnight to reach an OD_600_ of four the following morning. Cultures were pelleted at 1500*g* for 2 min and resuspended in 0.5 mL buffer A (15 mM Tris at pH 7.5, 80 mM KCl, 0.1 mM EGTA, 0.2 mM spermine, 0.5 mM spermidine, 1× cOmplete EDTA-free protease inhibitors [Roche, one tablet per 50 mL buffer], and 1 mM PMSF) and then transferred to 2 mL 96-well plates (Thermo Fisher Scientific). Cells were washed twice in 1 mL buffer A. Next, the cells were resuspended in 150 µL buffer A containing 0.1% digitonin, transferred to an Eppendorf 96-well plate (Eppendorf), and incubated for 5 min at 30°C for permeabilization. Next, we added CaCl_2_ to a final concentration of 2 mM to activate the MNase and incubated it for 30 sec. The CaCl_2_ treatment was stopped by adding an equal volume of stop buffer (400 mM NaCl, 20 mM EDTA, 4 mM EGTA, and 1% SDS) to the cell suspension. After this, the cells were treated with Proteinase K (0.5 mg/mL) for 30 min at 55°C. An equal volume of phenol–chloroform (pH 8; Sigma-Aldrich) was added, vigorously vortexed, and centrifuged at 17,000*g* for 15 min to extract DNA. The DNA was precipitated after the extraction with 3 volumes of cold 96% EtOH, 45 mg GlycoBlue, and 20 mM sodium acetate for >1 h at –80°C. Next, the tubes were centrifuged (17,000*g*, 10 min at 4°C); the supernatant was removed; and the DNA pellet was washed with 70% EtOH. The DNA pellets were dried and resuspended in 30 µL RNase A solution (0.33 mg/mL RNase A in Tris-EDTA [TE] buffer [10 mM Tris and 1 mM EDTA]) and treated for 20 min at 37°C. DNA cleanup was performed using SPRI beads (Ampure XP, Beckman Coulter) to enrich small DNA fragments from the MNase DNA cuts and remove large DNA fragments that might result from spontaneous DNA breaks. First, a reverse SPRI cleanup was performed by adding 0.8× (24 µL) SPRI beads, followed by 5 min incubation at room temperature (RT). The supernatant was collected, and the remaining small DNA fragments were purified by adding additional 1× (30 µL) SPRI beads and 5.4× (162 µL) isopropanol and incubating for 5 min at RT. The beads were washed twice with 85% EtOH, and finally, DNA was eluted in 30 µL of 0.1× TE buffer.

#### Mammalian

The ChEC protocol was adapted from that of yeast as described above. Prior to the experiment, cells were washed three times with buffer A (20 mM Hepes buffer [Biological Industries 03-025-1B], 110 mM potassium acetate, 5 mM sodium acetate, 0.2 mM spermine, 0.5 mM spermidine, 1× cOmplete EDTA-free protease inhibitors [Roche, one tablet per 50 mL buffer], 1 mM PMSF, and 1.5 mM EGTA). Cells were permeabilized using 250 µL of buffer A without EGTA and 0.05% digitonin for 5 min. Next, 50 µL CaCl_2_ was added to a final concentration of 2 mM to activate the MNase and was incubated for 2 min. The CaCl_2_ treatment was stopped by adding 100 µL of stop buffer (800 mM NaCl, 40 mM EDTA, 8 mM EGTA, and 40% SDS) to the cell suspension. After this, the cells were treated with Proteinase K (0.5 mg/mL) for 1 h at 55°C. An equal volume of phenol–chloroform (pH 8; Sigma-Aldrich) was added, vigorously vortexed, and centrifuged at 17,000*g* for 15 min to extract DNA. The DNA was precipitated after the extraction with 2.5 volumes of cold 96% EtOH, 45 mg GlycoBlue, and 20 mM sodium acetate for >1 h at –80°C. Next, the tubes were centrifuged (17,000*g*, for 10 min at 4°C); the supernatant was removed; and the DNA pellet was washed with 70% EtOH. The DNA pellets were dried and resuspended in 30 µL RNase A solution (0.33 mg/mL RNase A in Tris-EDTA [TE] buffer [10 mM Tris and 1 mM EDTA]) and treated for 40 min at 37°C. DNA cleanup was performed using SPRI beads (Ampure XP, Beckman Coulter) to enrich small DNA fragments from the MNase DNA cuts and remove large DNA fragments that might result from spontaneous DNA breaks. First, a reverse SPRI cleanup was performed by adding 0.8× (24 µL) SPRI beads, followed by 5 min incubation at RT. The supernatant was collected, and the remaining small DNA fragments were purified by adding additional 1× (30 µL) SPRI beads and 5.4× (162 µL) isopropanol and incubating for 5 min at RT. The beads were washed twice with 85% EtOH, and finally, DNA was eluted in 30 µL of 0.1× TE buffer.

### ChEC-seq high-throughput sequencing library preparation

Library preparation was performed as previously described (Skene and Henikoff 2017), with modifications. Following RNase treatment and reverse SPRI cleanup, the DNA fragments served as an input to an end-repair and A-tailing (ERA) reaction. A 5.4 µL ERA reaction was prepared (1× T4 DNA ligase buffer [NEB], 0.5 mM dNTPs, 0.25 mM ATP, 2.75% PEG 4000, 6 U T4 PNK [NEB], 0.5 U T4 DNA polymerase [Thermo Fisher Scientific], and 0.5 U Taq DNA polymerase [Bioline]) and added to 14.6 µL of each sample and incubated for 20 min at 12°C, 15 min at 37°C, and 45 min at 58°C in a thermocycler. After the ERA reaction, reverse SPRI cleanup was performed by adding 0.5× (10 µL) SPRI beads (Ampure XP, Beckman Coulter). The supernatant was collected, and the remaining small DNA fragments were purified with additional 1.3× (26 µL) SPRI beads and 5.4× (108 µL) isopropanol. After washing with 85% EtOH, small fragments were eluted in 17 µL of 0.1× TE buffer; 16.4 µL elution was taken into 40 µL ligation reaction (1× quick ligase buffer [NEB], 4000 U quick ligase [NEB], and 6.4 nM Y-shaped barcode adaptors with T-overhang) and incubated for 15 min at 20°C in a thermocycler. After incubation, the ligation reaction was cleaned by performing a double SPRI cleanup. First, a regular 1.2× (48 µL) SPRI cleanup was performed and eluted in 30 µL 0.1× TE buffer. Then instead of separating the beads, an additional SPRI cleanup was performed by adding 1.3× (39 µL) HXN buffer (2.5 m NaCl, 20% PEG 8000) and final elution in 24 µL 0.1× TE buffer; a 23 µL elution was taken into a 50 µL enrichment PCR reaction (1× Kappa HIFI [Roche], 0.32 µM barcoded Fwd primer, and 0.32 µM barcoded Rev primer) and incubated for 45 sec in 98°C, 16 cycles of 15 sec in 98°C and 15 sec in 60°C, and a final elongation step of 1 min at 72°C in a thermocycler.

The final libraries were cleaned using 1.1× (55 µL) SPRI and eluted in 15 µL 0.1× TE buffer. Library concentration and size distribution were quantified by Qubit (Thermo Fisher Scientific) and TapeStation (Agilent), respectively. For multiplexed high-throughput sequencing, all barcoded libraries were pooled in equal amounts, and the final pool was diluted to 2 nM and sequenced on NovaSeq 6000/ NovaSeq X (Illumina). Sequence parameters were read1, 61 nt; Index1, 8 nt; Index2, 8 nt; Read2, 61 nt.

### Western blotting

NMuMG cells expressing full-length, ΔLBD, and DBD mutants were harvested and lysed in RIPA buffer supplemented with DTT and proteinase inhibitors. Lysates were incubated on ice for 15 min and cleared by centrifugation at 14,000*g* for 30 min at 4°C, and the protein concentration was determined using a BCA assay. Protein loading was calculated using a reference sample, and samples were mixed with sample buffer, denatured for 5 min at 95°C, resolved by SDS–PAGE, and transferred onto nitrocellulose membranes. Membranes were blocked in Tris-buffered saline containing 0.1% Tween-20 (TBST) supplemented with 5% bovine serum albumin (BSA) for 1 h at RT. All subsequent washes and antibody incubations were performed using TBST containing 5% BSA. Membranes were incubated overnight at 4°C with primary antibodies (mouse anti-FLAG tag, 1:4000, Sigma-Aldrich F3165; rabbit anti-tubulin, 1:2000, Abcam ABT170), applied simultaneously at the indicated dilutions. Following primary antibody incubation, membranes were washed three times for 5 min each in TBST with 5% BSA and then incubated for 1 h at RT with species-specific HRP-conjugated secondary antibodies. Membranes were washed again three times in TBST with 5% BSA, and proteins were visualized using enhanced chemiluminescence (ECL) reagents. Signals were detected using a digital imaging system.

### Computational analyses

NGS data processing pipelines were built using Snakemake (Mölder et al. 2021). All computational analyses were conducted in Python using Jupyter (Kluyver et al. 2016) notebooks and custom scripts. Data were stored in data frames utilizing the Pandas and Polars libraries. Statistical analyses were performed with NumPy (Harris et al. 2020) and SciPy (Virtanen et al. 2020). For general plotting, Matplotlib (Hunter 2007) and seaborn (Waskom 2021) were used, whereas sequence logos of motifs were visualized using the LogoMaker package. Genomic range processing in Python was performed with PyRanges (Stovner and Saetrom 2020) in conjunction with pybigWig and pysam. Biological sequence analyses were conducted using Biopython (Cock et al. 2009).

### ChEC-seq NGS data processing

Raw reads from ChEC-seq libraries were demultiplexed using bcl2fastq (Illumina), and adaptor dimers and short reads were filtered out using cutadapt (Martin 2011) with the following parameters: “—O 10 –pair-filter = any –max-n 0.8 –action = mask.” Filtered reads were subsequently aligned to the *S. cerevisiae* genome R64-1-1, murine genome mm10, and human genome hg38 using Bowtie 2 (Langmead et al. 2012) with the options “‐‐end-to-end ‐‐trim-to 40 ‐‐very-sensitive.” The genome coverage of fully aligned read pairs was calculated with GenomeCoverage by BEDTools (Quinlan and Hall 2010) using the parameters “-d –5 –fs 1,” resulting in the position of the fragment ends, which correspond to the actual MNase cutting sites. For yeast experiments, the reads were normalized to 10^7^, excluding the ribosomal locus on the XII Chromosomes and the CUP1/2 gene regions.

### Data normalization and filtering for single-nucleotide cuts analysis (mammalian)

Raw signal data were processed through normalization and filtering steps to ensure data quality. First, blacklisted regions and signals from mitochondrial and Y Chromosomes (for the NMuMG cell line and the mitochondrial chromosome for HepG2) were removed. The data were then expanded so that each entry in the bedGraph would cover only 1 nt and normalized to 10 × 10^6^, allowing comparisons across samples. Nucleotides with fewer than three (unnormalized) cuts were excluded from the analysis.

### Yeast promoter definition

Promoters were defined only for genes with an annotated transcript, as described previously (Brodsky et al. 2020; Jonas et al. 2023). The length of each promoter was defined from the TSS with a minimal length of 700 or as a length to the regions of another transcript. The promoters of subtelomeric genes were excluded from the analyses.

### Yeast motif binding analyses

To analyze the signal on motifs bound by SRs in the S. *cerevisiae* genome, we first analyzed the yeast promoters on occurrences of the native (SR) motif and secondary (FOX, AP-1, half-site, KLF) motifs using FIMO (MEME suite) (Bailey et al. 2015). The signal from the middle of the motif ±100 bp residing in promoters was then extracted from the normalized data, and the top 10% of the motifs was plotted as a mean enrichment profile.

### Peak calling and filtering

For mammalian ChEC-seq experiments, peak calling was performed using MACS3 (Zhang et al. 2008) with parameters --format BAMPE -g hs for hg38 or -g mm for mm10, along with the options –no-model –no-lambda ‐‐keep-dup all. The called peaks were subsequently filtered using a custom Python script to remove peaks overlapping blacklisted regions. The peaks were then combined between the replicates using the IDR procedure with ChIP-R (Newell et al. 2021). To refine peak selection, signal intensity over peaks was compared against local flanking regions. The fold-change was calculated as the ratio of peak signal intensity to the mean signal in the flanking regions of the same size. To ensure that only the high-confidence peaks were taken, peaks with fold-change ≥ 10 were retained.

### bigWig normalization and averaging

bigWig coverage files were generated from BAM alignments using bamCoverage of deepTools (Ramírez et al. 2016), normalizing by counts per million. The signal between repeats was then averaged using bigWigAverage.

### De novo motif enrichment

High-confidence peaks were analyzed with HOMER v2 (Heinz et al. 2010) with default parameters to find the enriched motifs.

### Peak clustering

For binding similarity analysis, peaks of presented samples were merged and clustered using the PyRanges package.

### Motif annotation and enrichment analysis

To annotate the motifs present in each peak, we converted the analyzed peak set to a FASTA file using the BEDTools getFASTA program and used the FIMO program from the MEME suite to find the respective motifs in peaks. To check for the enrichment of the motifs in the peaks, we first converted the peaks to FASTA files as described above and used SEA (MEME suite) to extract the enrichment of the respective motifs inside peaks.

### Peak binding similarity analysis

For each analyzed sample, the signal of the final (individual or clustered set of peaks) was extracted as follows. We first defined the center of the peak as a middle between the peak borders; then using the pybigWig package, the signal ±300 bp from the middle was used as a sum signal for the respective peak. To broadly analyze the similarity in binding patterns between the samples, we calculated the Pearson's *r* correlation coefficient between the sum signal of the peaks. We visualized it as heat maps. We first transformed the sum signal on the peak matrix using *Z*-score normalization to visualize the differentially bound peaks. We defined the differentially bound peaks as those bound with a *Z*-score higher than three in the target sample and lower than two in the compared ones; the signal on the resulting peaks was plotted ±1 kb from the defined middle of the peak.

### Interread distance calculation

To calculate per-nucleotide cut distances, we first extracted the 5′ ends of aligned reads using “SAMtools view -h -f 66” coupled with “BEDTools genomecov -bg –5 –fs 1.” We then expanded the data in a way that each entry in the bedGraph would correspond to a single nucleotide and measured the distances between these cut sites across the analyzed chromosomes. Empirical cumulative distribution fractions (ECDFs) of the interread distances were generated at various read count thresholds to assess sequencing consistency across all replicates of the sample.

### Coverage fraction analysis

To evaluate sequencing coverage distribution, we calculated the fraction of base pairs with (nonnormalized) MNase cuts that exceeded a given threshold. The cumulative fraction of the covered base pairs was computed across a range of coverage values. These fractions were then plotted against coverage thresholds, providing a comparative assessment of sequencing depth across different conditions.

### Footprint length analysis

To assess TF footprinting, we identified the top 10% of bound motif locations based on the sum of signal intensity within ±100 bp from the motif center. For each motif within the analyzed peak set, an average signal profile was generated. From this profile, the five highest-intensity positions were selected as unprotected from cleavage, representing accessible regions, and the distribution of reads at these unprotected sites was used as a reference for nonprotected regions.

To quantify footprinting, the read distributions at all other positions were compared to the reference using the Mann–Whitney *U* test. Footprint lengths were then determined by analyzing signal intensity drop-offs around the peak signal of the transformed *P*-values. To ensure accurate peak detection, signal profiles were interpolated to generate a dense distribution. At predefined percentage thresholds (ranging from 5% to 100% of peak intensity), the genomic positions at which the signal intensity dropped below the threshold were identified on both sides of the peak. The footprint length was defined as the distance between these two positions.

Finally, footprint length distributions between the 5% and 50% thresholds were visualized using violin plots.

### Peak annotations

To annotate the distribution of peaks across different *cis*-regulatory regions, we used annotations obtained from SCREEN (The ENCODE Project Consortium et al. 2020).

## Data access

All raw and processed sequencing data generated in this study have been submitted to the NCBI Gene Expression Omnibus (GEO; https://www.ncbi.nlm.nih.gov/geo/) under accession number GSE293974. Custom Python scripts and notebooks used to produce the figures are available at Zenodo (https://doi.org/10.5281/zenodo.19454463) and as Supplemental Code.

## Supplemental Material

Supplement 1

Supplement 2

Supplement 3
