# Supplementary material for: Spatially resolved profiling of steroid nuclear receptors reveals a role for the disordered N-terminal domains in genome targeting and AP-1 interaction
Source: Genome Res. 2026 Jul;36(7):1386–402. doi: 10.1101/gr.280896.125 (PMC13322194; doi:10.1101/gr.280896.125)
Supplement: Supplement 1 [file Supplemental_Figures.pdf]

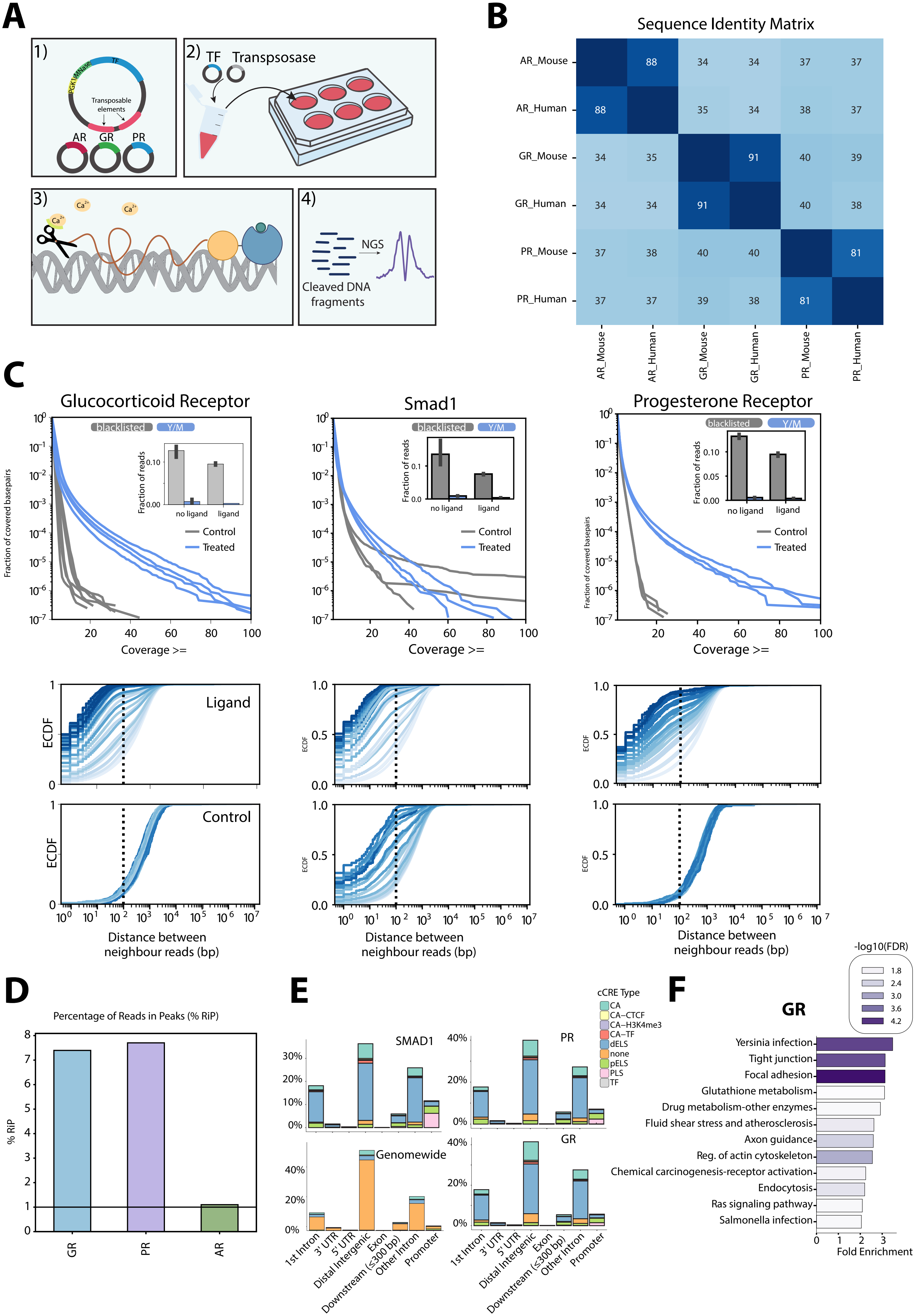

A
1)
PGK1/MNase
TF
Transposable elements
AR GR PR
2) TF Transpsosase
3)
Ca2+
4)
NGS
Cleaved DNA fragments
B
Sequence Identity Matrix
AR_Mouse AR_Human GR_Mouse GR_Human PR_Mouse PR_Human
C
Glucocorticoid Receptor
Smad1
Progesterone Receptor
blacklisted Y/M
Fraction of reads
no ligand ligand
Control
Treated
Fraction of covered basepairs
Coverage >=
Ligand
Control
ECDF
Distance between neighbour reads (bp)
D
Percentage of Reads in Peaks (% RiP)
% RiP
GR PR AR
E
SMAD1
PR
Genomewide
GR
cCRE Type
CA
CA–CTCF
CA–H3K4me3
CA–TF
dELS
none
pELS
PLS
TF
1st Intron
3' UTR
5' UTR
Distal Intergenic
Exon
Downstream (≤300 bp)
Other Intron
Promoter
F
GR
-log10(FDR)
Yersinia infection
Tight junction
Focal adhesion
Glutathione metabolism
Drug metabolism-other enzymes
Fluid shear stress and atherosclerosis
Axon guidance
Reg. of actin cytoskeleton
Chemical carcinogenesis-receptor activation
Endocytosis
Ras signaling pathway
Salmonella infection
Fold Enrichment

Supplementary Figure 1: Using ChEC-seq for mapping Transcription factors (TFs) binding locations across mammalian genomes

(A). Adapting ChEC-seq for use with mammalian cell lines: (Scheme) the ORFs of SRs were integrated into PiggyBack expression plasmid downstream to PGK1 and MNase (methods), the resulting construct was transfected into the cell line. On the day of the experiment, the short Ca2+ pulse (2 min, methods) activated MNase, which cuts proximal DNA. The fragments were collected, sequenced, and mapped to the genome (methods).
(B). SR sequences are similar between humans and mice: Shown is the percentage identity matrix (calculated by Clustal Omega (Sievers and Higgins 2018)) between the human and mouse SR sequences. Note the high similarity between the same TF homologs.
(C). ChEC-seq profiles of stimulated SRs are distinguished by the distribution of cleaved sites: Shown are the distributions of per-nucleotide coverage for hormone-stimulated and non-stimulated TFs (left, blue and gray lines, all repeats are included), and the distribution of distances between cleavage sites (bottom, with cleavage events grouped by read coverage indicated by color intensity, methods). Note that cleavage sites are sparsely distributed in the control sample but tend to accumulate at the same or adjacent reads in the activated ones. The fraction of reads that map to the blacklisted regions and the Y or Mitochondrial chromosomes is shown in the insets. Note for SMAD1, there is also an observed signal in the control since its activating ligand was also present in freshly replenished media.
(D-F). Analyzed peaks localize to the known previously defined regulatory regions: Shown in (D) is the percentage of analyzed reads inside the high confidence peaks for 3 SRs (methods). The vertical black line is on the 1% mark (common experiment success criteria as defined by ENCODE for ChIP-seq). The distribution of peaks across different genomic regions and kinds of *Cis*-Regulatory Regions (CRE) as defined by SCREEN (Moore et al. 2026) for SMAD1, PR, all genome, and GR is presented in (E), and the KEGG annotation for genes linked to peaks of GR (T-gene)(Bailey et al. 2015) is shown in (F).

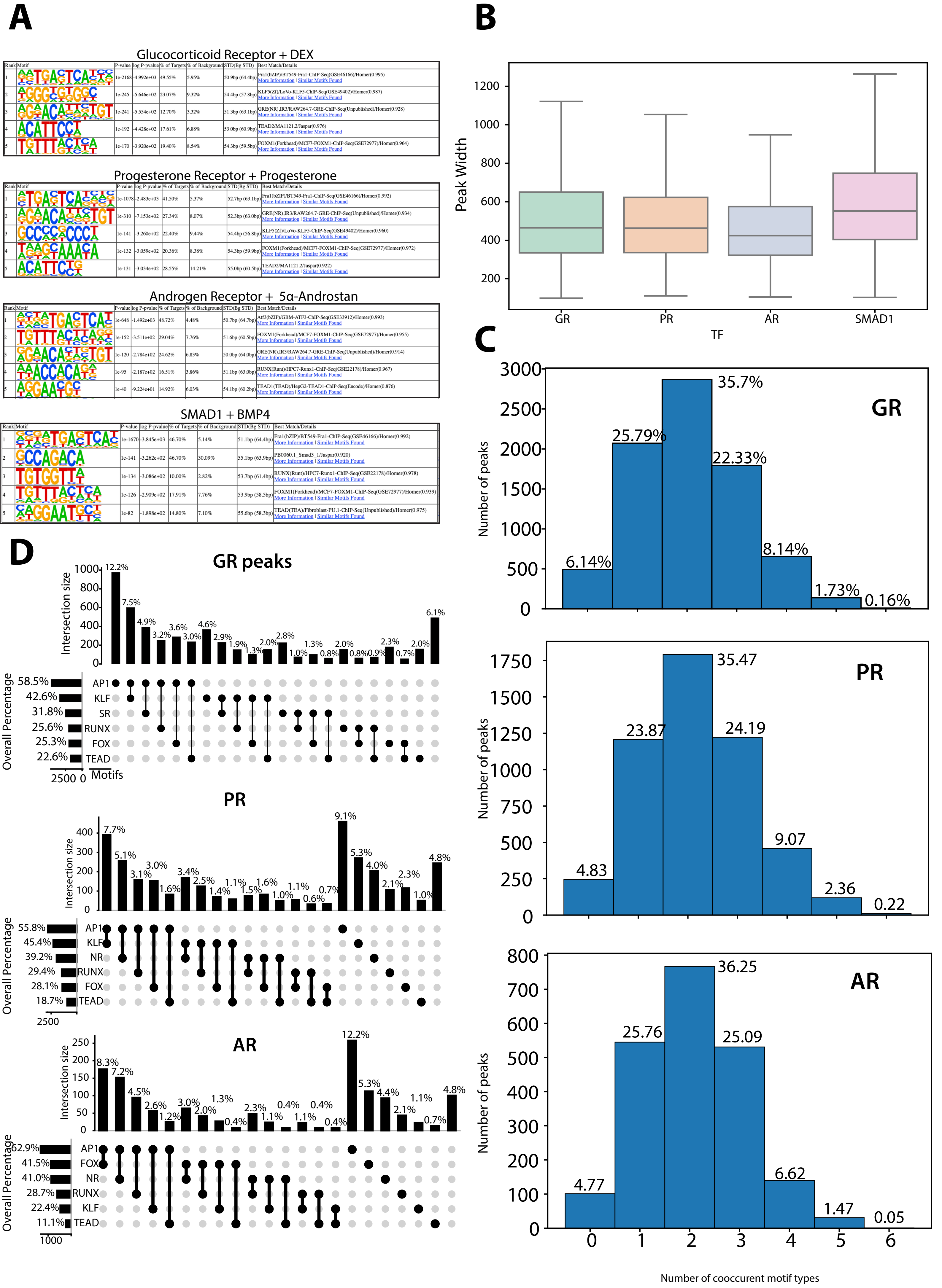
A
Glucocorticoid Receptor + DEX
Progesterone Receptor + Progesterone
Androgen Receptor + 5α-Androstan
SMAD1 + BMP4
Rank Motif P-value log P-pvalue % of Targets % of Background STD(Bg STD) Best Match/Details
B
Peak Width
GR
PR
AR
SMAD1
TF
C
GR
PR
AR
Number of peaks
Number of cooccurent motif types
D
GR peaks
PR
AR
Intersection size
Overall Percentage
Motifs
AP1
KLF
SR
NR
RUNX
FOX
TEAD

Supplementary Figure 2: Analyzed TFs localize to the canonical and interacting motifs

(A). De-novo motif enrichment of TFs: Presented in (A) are the de-novo motif enrichment results of the high confidence peaks of GR, PR, AR, and SMAD1 (top 5 enriched motifs are shown, methods).
(B-D). Analyzed peaks contain 1-3 enriched motifs: Shown in (B) are the distributions of peak sizes for the indicated TFs, with the distribution of a number of motif types in each peak for the 3 SRs shown in (C). Presented in (D) is a more detailed analysis of motif pairs/individuals in the analyzed peaks.

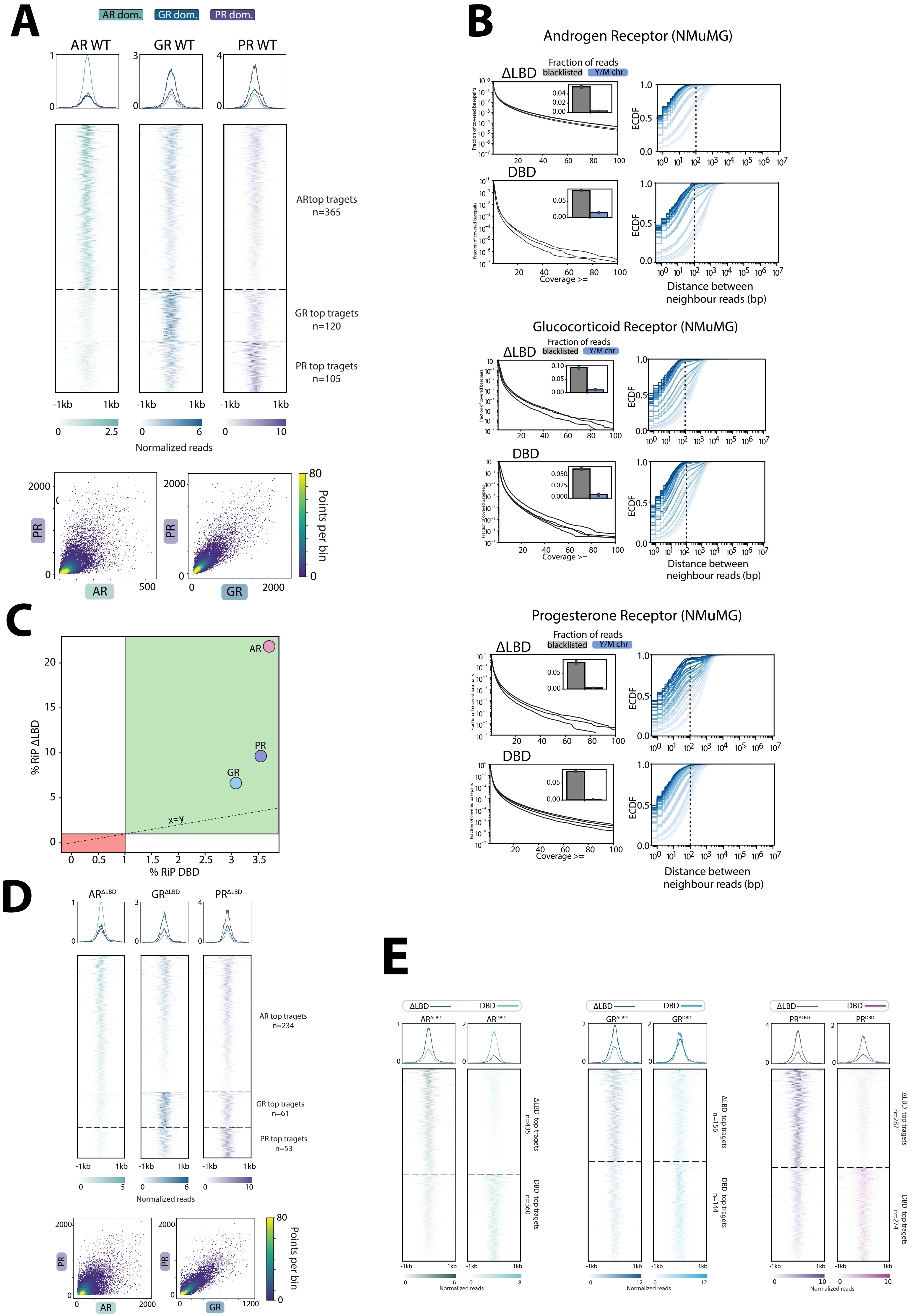
A
AR dom.
GR dom.
PR dom.
AR WT
GR WT
PR WT
ARtop tragets n=365
GR top tragets n=120
PR top tragets n=105
-1kb
1kb
Normalized reads
PR
AR
GR
Points per bin
B
Androgen Receptor (NMuMG)
Fraction of reads
blacklisted
Y/M chr
ΔLBD
DBD
Fraction of covered basepairs
Coverage >=
ECDF
Distance between neighbour reads (bp)
Glucocorticoid Receptor (NMuMG)
Progesterone Receptor (NMuMG)
C
% RiP ΔLBD
% RiP DBD
x=y
D
AR top tragets n=234
GR top tragets n=61
PR top tragets n=53
E
ΔLBD top tragets n=435
DBD top tragets n=360
ΔLBD top tragets n=156
DBD top tragets n=144
ΔLBD top tragets n=287
DBD top tragets n=274

Supplementary Figure 3: Profiling of SR mutants using ChEC-seq in the NMuMG cell line

(A). Binding peaks of the 3 SRs differ substantially: the intra-mutant binding comparison for differentially bound peaks (methods). The average profiles of the defined peak groups are shown on top of each heatmap, and the corresponding scatter plots are shown on the bottom.
(B-C). ΔLBD and DBD mutants of SR meet QC criteria defined for the WT proteins: Shown in (A) are previously defined QC metrics for ΔLBD and DBD mutants (presentation as in Fig. S1C). Presented in (B) is the comparison of the percentage of reads in high-confidence peaks comparing the DBD and ΔLBD. The diagonal line is x=y, the red box encloses the 1% region, and the green box is>1%.
(D). Binding peaks of the 3 ΔLBD mutants differ substantially, as in (A) for ΔLBD mutants.
(E). DBD mutants shift binding preference: As in (A), comparing the ΔLBD and DBD mutants.

A

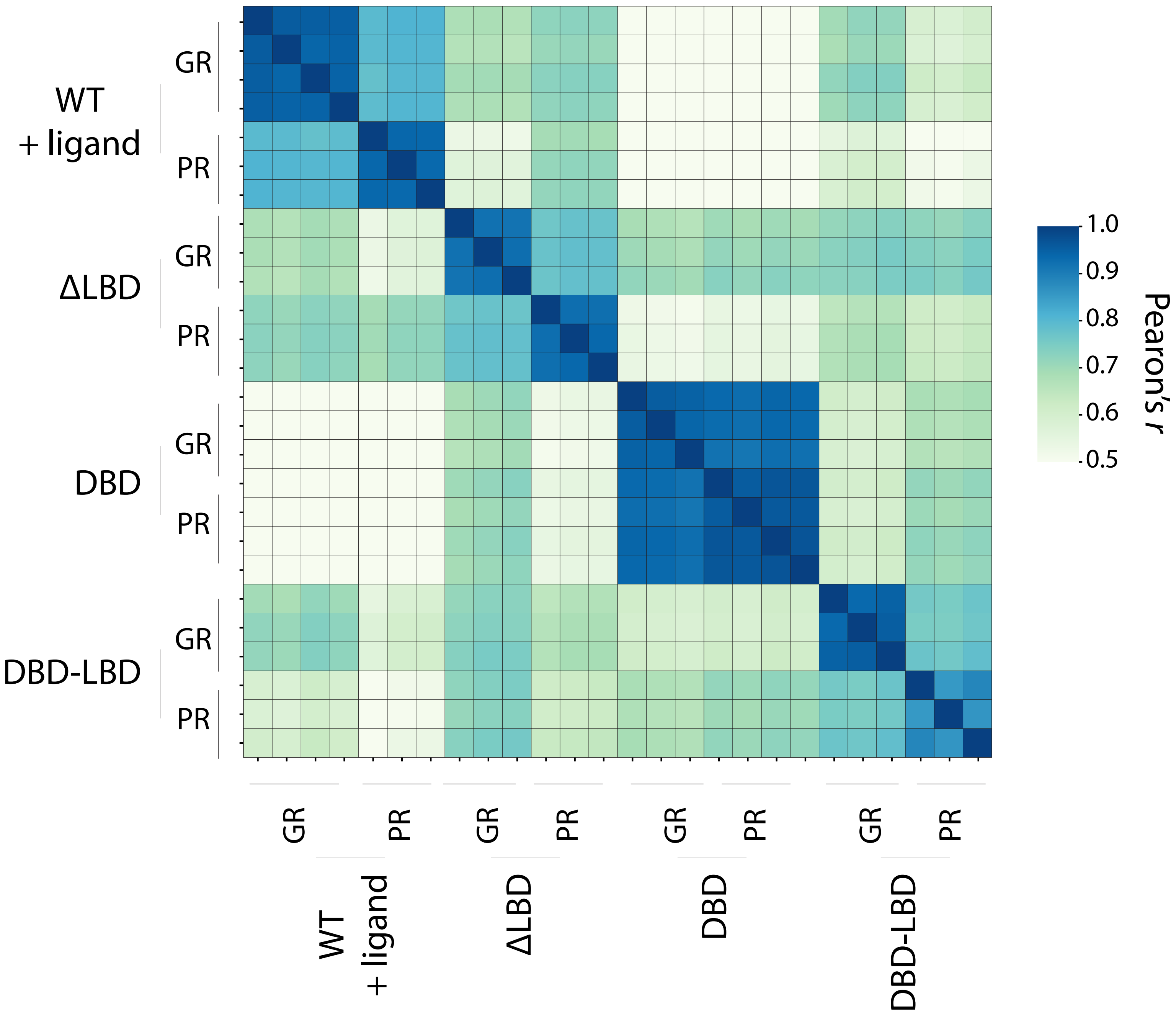

Supplementary Figure 4: Profiling the effects of the LBD on the binding of the SRs

Shown are the correlations (Pearson's r) between the indicated SRs and their mutants. All peaks of the respective SRs and mutants were merged into a consensus peak set, and the sum signal on the peak (defined as +/- 300 bp from the middle of the peak) was calculated for each repeat. All repeats are shown.

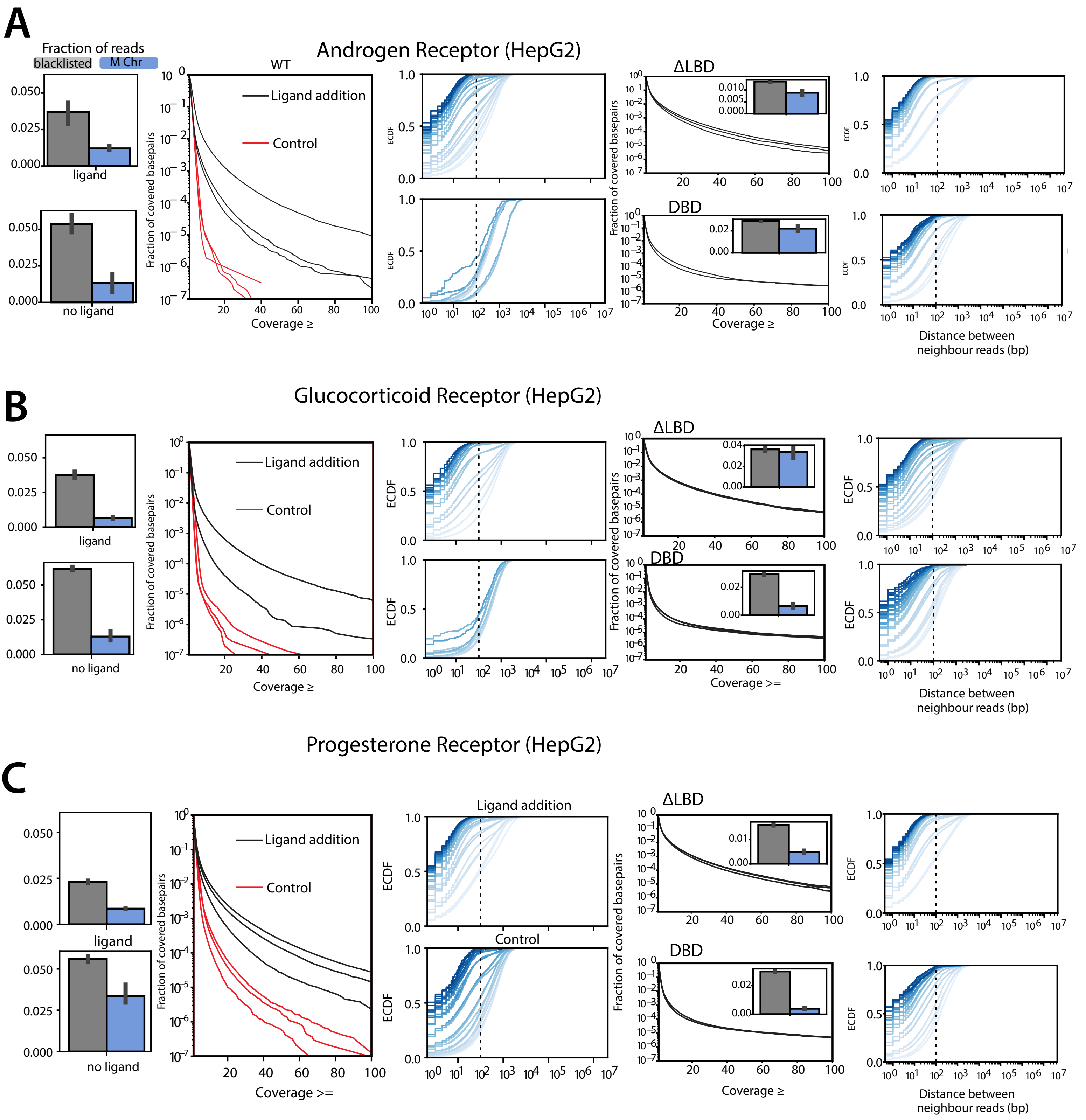
A
Androgen Receptor (HepG2)
Fraction of reads
blacklisted
M Chr
ligand
no ligand
WT
Ligand addition
Control
Fraction of covered basepairs
Coverage ≥
ECDF
ΔLBD
DBD
Distance between neighbour reads (bp)
B
Glucocorticoid Receptor (HepG2)
ligand
no ligand
Ligand addition
Control
Fraction of covered basepairs
Coverage ≥
ECDF
ΔLBD
DBD
Coverage >=
Distance between neighbour reads (bp)
C
Progesterone Receptor (HepG2)
ligand
no ligand
Ligand addition
Control
Fraction of covered basepairs
Coverage >=
Ligand addition
Control
ECDF
ΔLBD
DBD
Coverage ≥
Distance between neighbour reads (bp)

Supplementary Figure 5: Profiling of SRs and their mutants in the HepG2 cell line

(A-C). The data for SRs and their variants in the HepG2 cell line meet previously defined QC criteria: Shown are previously defined QC metrics for all performed experiments in the HepG2 cell line. Note the high enrichment of signal also for the PR control (no progesterone addition), pointing at the activity of this TF also in the absence of progesterone hormone in the HepG2 cell line (presentation as in Fig. S1C).

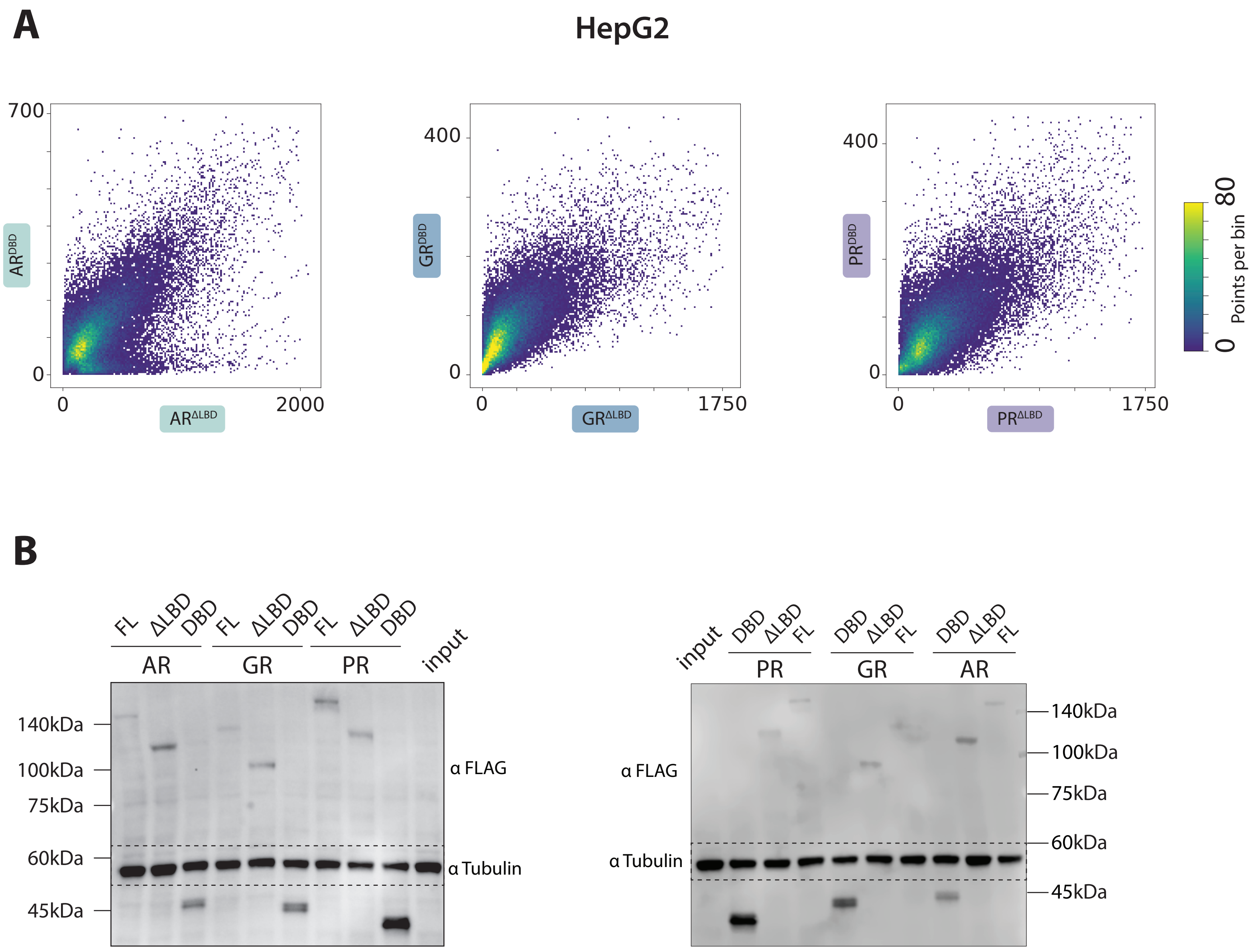
A
HepG2
700
0
AR^DBD
AR^ΔLBD
0
2000
400
0
GR^DBD
GR^ΔLBD
0
1750
400
0
PR^DBD
PR^ΔLBD
0
1750
80
0
Points per bin
B
FL
ΔLBD
DBD
FL
ΔLBD
DBD
FL
ΔLBD
DBD
AR
GR
PR
input
140kDa
100kDa
75kDa
60kDa
45kDa
α FLAG
α Tubulin
input
DBD
ΔLBD
FL
DBD
ΔLBD
FL
DBD
ΔLBD
FL
PR
GR
AR
α FLAG
α Tubulin
140kDa
100kDa
75kDa
60kDa
45kDa

Supplementary Figure 6: Differences in SR binding profiles are not explained by LBD/ DBD preferences or a decrease in protein abundance of the DBD mutants

(A). Binding profiles of the SRs and their mutants were internally compared: Shown are the respective scatter plots between the DBD-only and ΔLBD mutants (99.8% of data is shown to remove the outliers, and the color indicates density) in the HepG2 cell line.
(B). Western blot of FLAG-tagged full-length FL, ΔLBD, and DBD mutants of AR, GR, and PR: Membranes were probed with anti-FLAG to detect SR mutants and with anti-α-tubulin as a loading control. Molecular-weight markers (kDa) are indicated on the left; “input” denotes whole-cell lysate. Two replicates of the Western blot are shown.

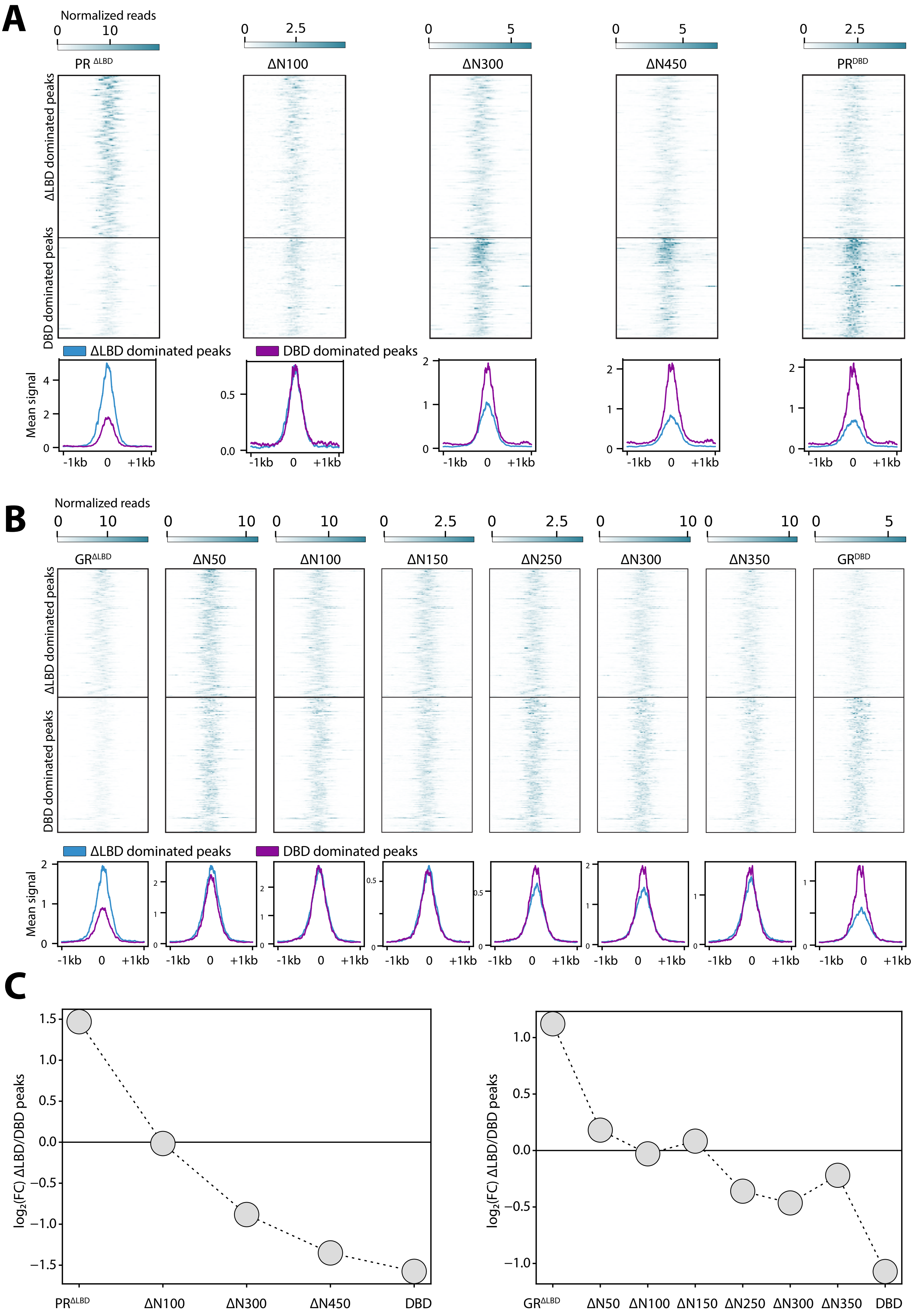

A
Normalized reads
PR$^{\Delta LBD}$
ΔN100
ΔN300
ΔN450
PR^{DBD}
ΔLBD dominated peaks
DBD dominated peaks
Mean signal
-1kb
0
+1kb
B
Normalized reads
GR$^{\Delta LBD}$
ΔN50
ΔN100
ΔN150
ΔN250
ΔN300
ΔN350
GR^{DBD}
ΔLBD dominated peaks
DBD dominated peaks
Mean signal
C
log$_2$(FC) ΔLBD/DBD peaks
PR$^{\Delta LBD}$
ΔN100
ΔN300
ΔN450
DBD
GR$^{\Delta LBD}$
ΔN50
ΔN100
ΔN150
ΔN250
ΔN300
ΔN350
DBD

Supplementary Figure 7: Multiple weak determinants spread across the SR NTD-sequence guide their binding specificity in mammalian genomes

(A-C). The SR binding profiles are gradually shifted with increasing NTD truncations: Shown in (A, B) is the gradual loss of binding at peaks upon NTD truncations (top panels, methods), as well as the mean signal (bottom panels). Log_2 fold-change of signal on the ΔLBD-dominated peaks vs. DBD-dominated peaks is shown (C, PR truncations-left, GR truncations-right).

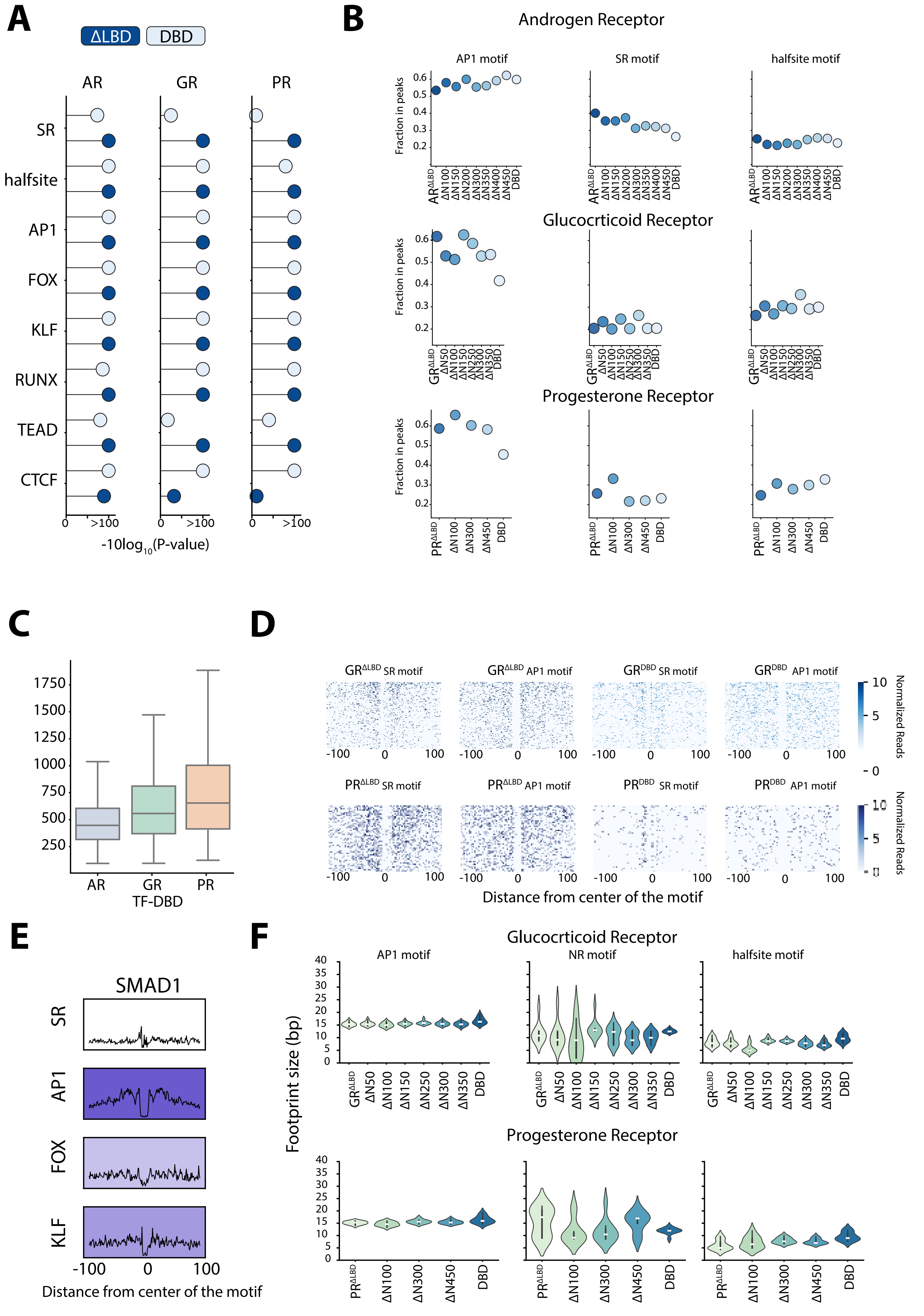
A
ΔLBD
DBD
AR
GR
PR
SR
halfsite
AP1
FOX
KLF
RUNX
TEAD
CTCF
0
>100
-10log10(P-value)
B
Androgen Receptor
AP1 motif
SR motif
halfsite motif
Fraction in peaks
Glucocrticoid Receptor
Progesterone Receptor
C
TF-DBD
D
Distance from center of the motif
Normalized Reads
E
SMAD1
SR
AP1
FOX
KLF
Distance from center of the motif
F
Glucocrticoid Receptor
AP1 motif
NR motif
halfsite motif
Footprint size (bp)
Progesterone Receptor

Supplementary Figure 8: Steroid Receptor DBDs Recapitulate ΔLBD Binding motifs and footprints signatures

(A). Binding peaks of SR DBD mutants lacking the NTD are enriched with the same motifs enriched in SR-bound peaks: Shown are the enrichment p-values of the indicated motifs (left) on the $SR^{\Delta LBD}$ (dark-blue) and SR^{DBD} (light-blue) mutants.
(B). *The fraction of motifs in peaks of SR truncation:* Analyzed here are the SR truncations and the fractions of AP1, SR, and SR half-site motifs inside the peak set of each respective mutant.
(C). *Peak sizes of SRs DBDs in consistent with the full-length SRs:* Shown are the distributions of peak sizes for the indicated SRs DBDs, presented as in Fig. S2B.
(D). Visualizing the SR and AP-1 footprint for ΔLBD, and DBD mutants of the GR and PR: Shown are the locations of cleavage sites (read ends) of the indicated factors at sequences surrounding the indicated motifs (methods). For this analysis, the top 10%-bound motifs within binding peaks were selected, ordered by the binding signal, and are shown as rows in this matrix, together with the ±100 bp surrounding regions (methods). Presented as in Fig. 5B.
(E). SMAD1 control binding profile shows no binding at the SR motif: The average profile of SMAD1 control over the indicated SR-enriched motifs.
(F). Footprint size signature for $GR^{\Delta LBD}$ and $PR^{\Delta LBD}$ and all their truncations: The estimated sizes of the cleavage-protected region (footprint size, methods) for the SR mutants for AP-1, SR, and half-site motifs. Presentation as in Fig. 5G.

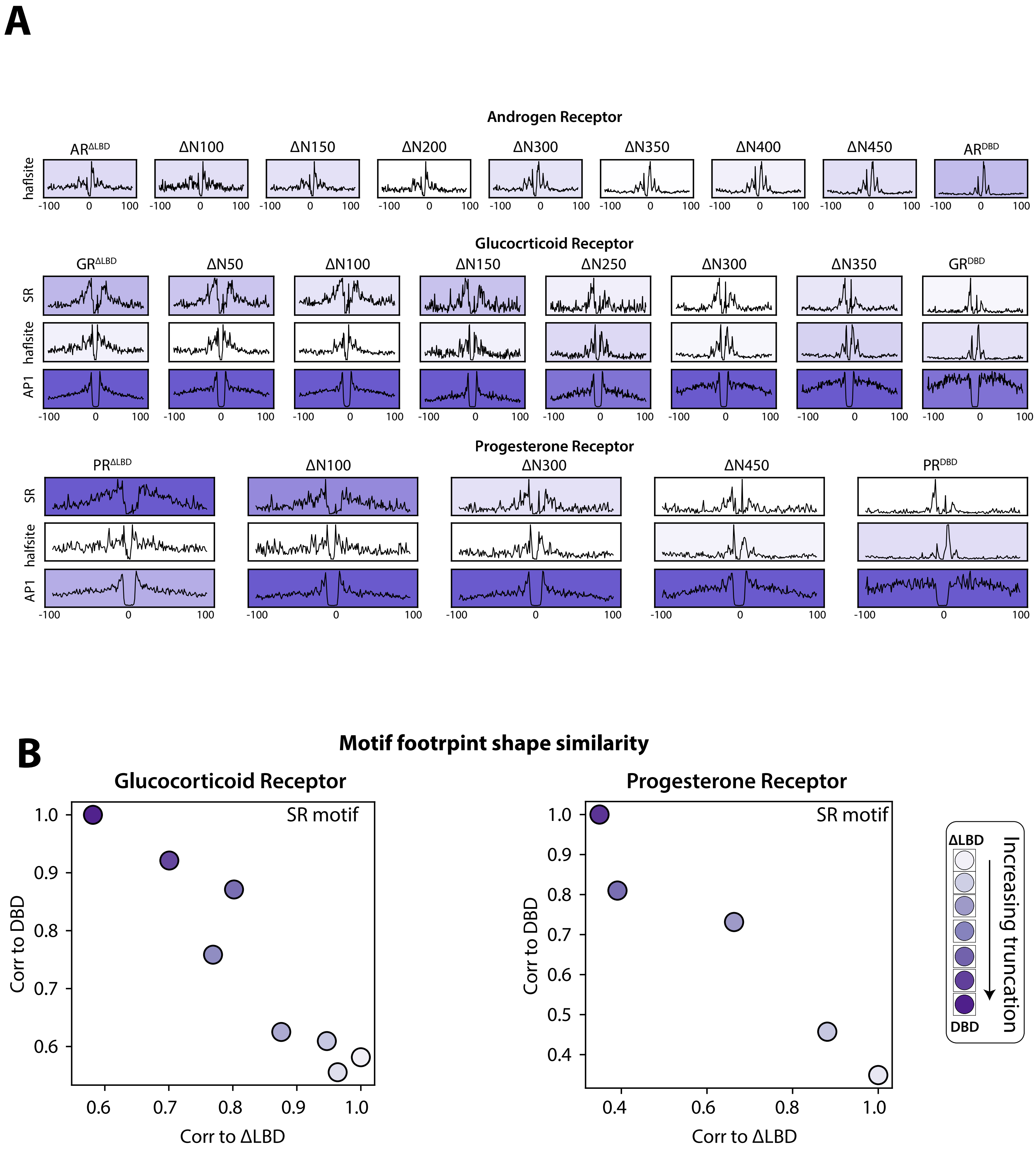

A
Androgen Receptor
AR^ΔLBD
ΔN100
ΔN150
ΔN200
ΔN300
ΔN350
ΔN400
ΔN450
AR^DBD
halfsite
-100 0 100
Glucocrticoid Receptor
GR^ΔLBD
ΔN50
ΔN100
ΔN150
ΔN250
ΔN300
ΔN350
GR^DBD
SR
halfsite
AP1
Progesterone Receptor
PR^ΔLBD
ΔN100
ΔN300
ΔN450
PR^DBD
B
Motif footrpint shape similarity
Glucocorticoid Receptor
Progesterone Receptor
SR motif
Corr to DBD
Corr to ΔLBD
1.0
0.9
0.8
0.7
0.6
0.5
0.4
ΔLBD
DBD
Increasing truncation

Supplementary Figure 9: Binding patterns of SRs gradually change with truncations

(A). SR truncations show a gradual change towards AP-1 dominance: The average profiles of the GR and PR truncations on the SR and AP-1 are shown. Presentation as in Fig. 5F for GR and PR. For AR, the half-site average profiles across truncations are presented.
(B). Truncations of the NTD change the footprint shape of the bound motifs: Correlation of SR motif signal to DBD reference motif (y-axis) versus ΔLBD reference motif (x-axis) for each truncation variant. Presentation, as in Fig. 5H for GR and PR.

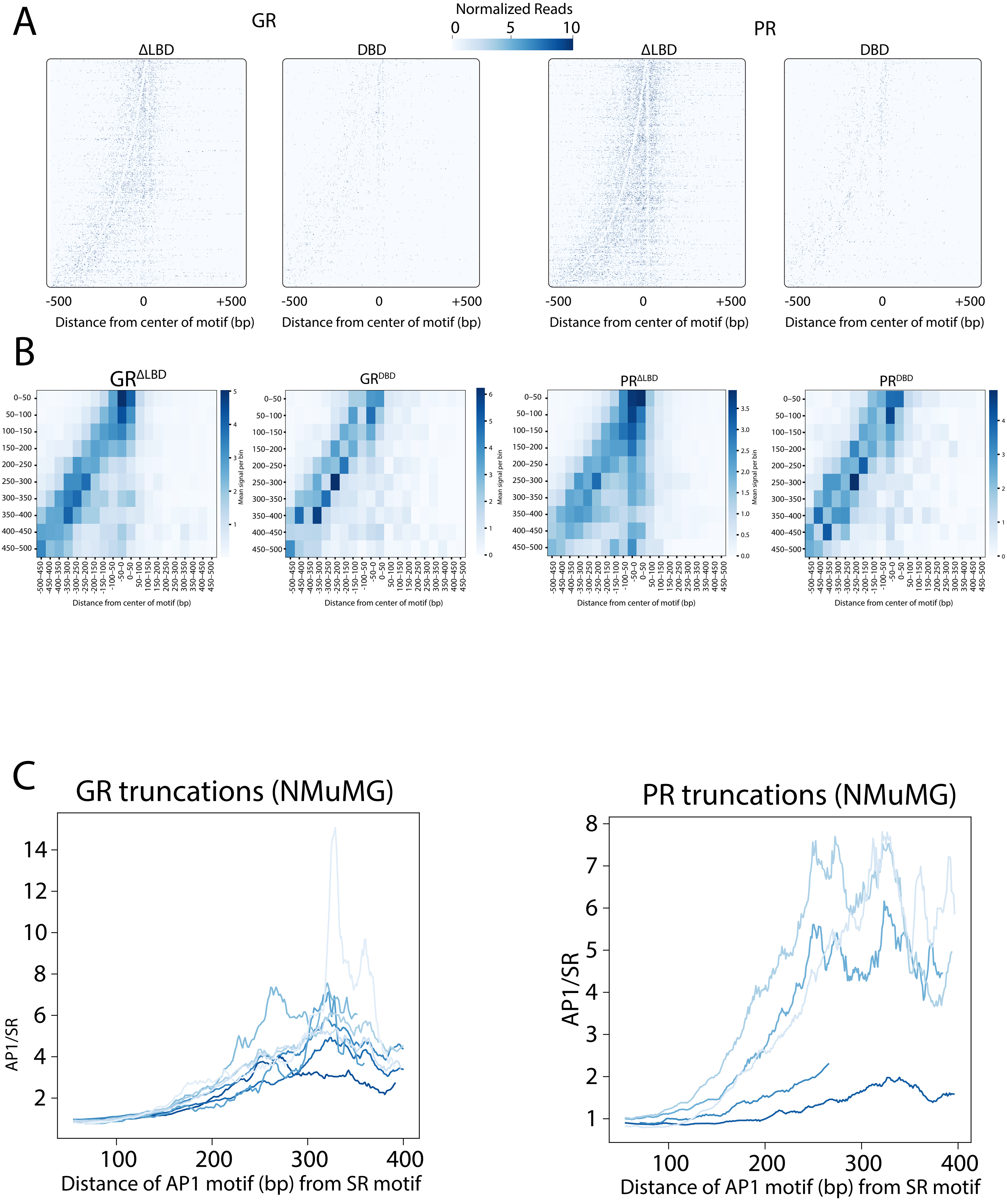

A
GR
PR
Normalized Reads
0
5
10
ΔLBD
DBD
ΔLBD
DBD
-500
0
+500
Distance from center of motif (bp)
B
GRΔLBD
GRDBD
PRΔLBD
PRDBD
0–50
50–100
100–150
150–200
200–250
250–300
300–350
350–400
400–450
450–500
Distance of AP1 motif from NR motif (bp)
Mean signal per bin
Distance from center of motif (bp)
C
GR truncations (NMuMG)
PR truncations (NMuMG)
AP1/SR
Distance of AP1 motif (bp) from SR motif

Supplementary Figure 10: Binding of the isolated SR DBD is partially dependent on the presence of the proximal AP1 motif

(A-C). SR^{DBD} binding at its canonical motif depends on proximity to the AP1 motif: Shown in (A) Are the genomic regions from the peaks containing both SR (canonical) and AP-1 motifs (non-canonical), aligned to the SR motif center and sorted by inter-motif distance, with AP-1 positioned to the left of the SR motif for GR and PR and their ΔLBD and DBD mutants. The heatmap shows MNase cleavage fragment ends, revealing protection patterns at both motif sites. Shown in (B) is the binding of GR and PR and their ΔLBD and DBD mutans binned by distance from SR center (x-axis, 50 bp) and AP1-to-SR spacing (y-axis, 50 bp). The color scale shows mean normalized reads per bin. The distance-dependent effect is summarized in C, showing the fold-change of the signal at the AP-1 vs. SR motifs as a function of the distances between motifs for the set of GR and PR truncations.

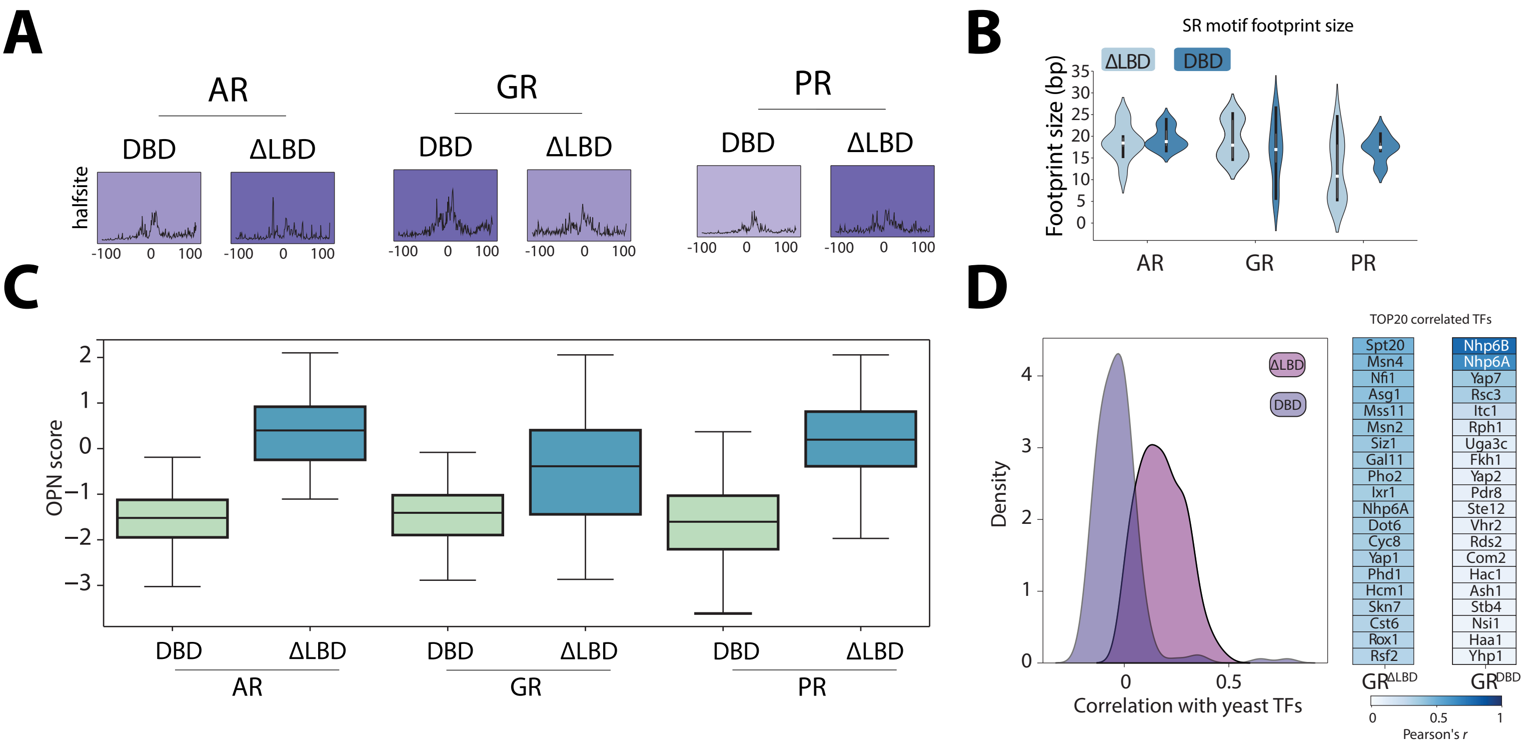
A
AR
GR
PR
DBD
ΔLBD
DBD
ΔLBD
DBD
ΔLBD
halfsite
-100 0 100
B
SR motif footprint size
ΔLBD
DBD
Footprint size (bp)
35
30
25
20
15
10
5
0
AR
GR
PR
C
OPN score
2
1
0
−1
−2
−3
DBD
ΔLBD
DBD
ΔLBD
DBD
ΔLBD
AR
GR
PR
D
Density
4
3
2
1
0
ΔLBD
DBD
0
0.5
Correlation with yeast TFs
TOP20 correlated TFs
Spt20
Msn4
Nfi1
Asg1
Mss11
Msn2
Siz1
Gal11
Pho2
Ixr1
Nhp6A
Dot6
Cyc8
Yap1
Phd1
Hcm1
Skn7
Cst6
Rox1
Rsf2
Nhp6B
Nhp6A
Yap7
Rsc3
Itc1
Rph1
Uga3c
Fkh1
Yap2
Pdr8
Ste12
Vhr2
Rds2
Com2
Hac1
Ash1
Stb4
Nsi1
Haa1
Yhp1
GRΔLBD
GRDBD
0
0.5
1
Pearson's r

Supplementary Figure 11: Using yeast as a 'cellular test tube' to study NTD effects on the genome binding preferences of SRs:

(A). Half-site motif binding by LBD-deleted SRs and their DBD mutants: The average profiles of the indicated factors around the half-site motif, background color as in Fig. 5D.
(B). Size footprint signature of mammalian LBD-deleted SRs and their DBD mutants remains unaffected in yeast: The size of the motif-related signature is summarized in the violin plot for
The three selected LBD-deleted SRs (ΔLBD).
(C). SR DBD mutants bind promoters with lower OPN scores: the distribution of OPN (Tirosh and Barkai 2008) scores of promoters bound by the indicated factors (target promoters defined as those with a *Z*-score > 3.5)
(D) *The binding profiles of mammalian SRs in yeast are unique:* the correlation of the indicated binding profiles with yeast TFs, displaying only the top-similar TFs of the ~95% profiles in our TF binding compendium.

Bailey, Timothy L., James Johnson, Charles E. Grant, and William S. Noble. 2015. "The MEME Suite." *Nucleic Acids Research* 43 (W1): W39-49.

Moore, Jill E., Henry E. Pratt, Kaili Fan, Nishigandha Phalke, Jonathan Fisher, Shaimae I. Elhajjajy, Gregory Andrews, et al. 2026. "An Expanded Registry of Candidate Cis-Regulatory Elements." *Nature*, January, 1–10.

Sievers, Fabian, and Desmond G. Higgins. 2018. "Clustal Omega for Making Accurate Alignments of Many Protein Sequences: Clustal Omega for Many Protein Sequences." *Protein Science* 27 (1): 135–45.

Tirosh, Itay, and Naama Barkai. 2008. "Two Strategies for Gene Regulation by Promoter Nucleosomes." *Genome Research* 18 (7): 1084–91.
